# Prospective associations of childhood metal mixtures with metabolic health in a Mexico City cohort

**DOI:** 10.1097/EE9.0000000000000535

**Published:** 2026-10-26

**Authors:** Carolyn Gigot, Pei Wen Tung, Haotian Wu, Guadalupe Estrada-Gutierrez, Mari Cruz Tolentino, Alejandra Cantoral, Julio Landero, Robert O. Wright, Martha M. Tellez-Rojo, Allison Kupsco

**Affiliations:** a Environmental Health Sciences, Columbia University, New York City, New York; b Environmental Health, Boston University, Boston, Massachusetts; c National Institute of Perinatology, Mexico City, Mexico; d Health Department, Iberoamerican University, Mexico City, Mexico; e Icahn School of Medicine at Mount Sinai, Environmental Medicine, New York City, New York; f National Institute of Public Health of Mexico, Center for Research in Nutrition and Health, Mexico City, Mexico.

## Abstract

**Background:**

Exposures to several metals and metalloids have been linked to impaired fasting glucose and insulin resistance that can progress to type 2 diabetes (T2D), which is increasing in prevalence among children and adolescents. However, the effects of metal mixtures on children’s metabolic health are less well characterized. We examined prospective associations of metals, individually and jointly, with metabolic parameters among children without T2D.

**Methods:**

We analyzed data from 422 children enrolled in the Programming Research in Obesity, Growth, Environment and Social Stressors (PROGRESS) cohort (Mexico City, 2007–2011). Eleven metals (arsenic, cadmium, chromium, cesium, lead, antimony, cobalt, copper, manganese, selenium, zinc) were measured in whole blood at the age 4-year visit. Fasting plasma glucose was measured at the 4-, 6-, 8-, and 10-year visits. Fasting insulin and homeostatic model assessment of insulin resistance (HOMA-IR) and β-cell function (HOMA-β) were determined at the 8- and 10-year visits. Associations were estimated using generalized additive mixed models, linear mixed models, and quantile g-computation.

**Results:**

All metal–outcome associations were linear. Selenium was positively associated with average fasting plasma glucose, insulin, and HOMA-IR; zinc was negatively associated with insulin and HOMA-IR. A quartile increase in the metal mixture was associated with 1.10-fold higher insulin (95% confidence interval [CI] = 1.01, 1.21) and 1.11-fold higher HOMA-IR (95% CI = 1.01, 1.22), with the largest contributions from selenium and arsenic.

**Conclusion:**

Early childhood metals, particularly selenium, zinc, and arsenic, were prospectively associated with insulin resistance, consistent with findings in adults. More work is needed to understand how early-life metal mixture exposures may affect metabolic health.

**What this study adds**

Metals such as arsenic, selenium, and zinc have been linked to diabetes and prediabetes, but evidence in children is limited and mostly cross-sectional. We measured 11 metals in blood at age 4 to 6 and followed fasting glucose and insulin resistance (HOMA-IR) through age 12. Higher selenium was associated with higher fasting glucose and insulin resistance over time, while higher zinc was associated with lower insulin resistance. The metal mixture—driven by selenium and arsenic—was associated with higher insulin resistance. Selenium and zinc are essential elements under homeostatic control, so associations may reflect altered cellular and tissue-level responses as well as exposure.

## Introduction

Diabetes is a leading cause of death and disability, with prevalence projected to increase to 1.31 billion people worldwide by 2050.^1^ More than 90% of cases in 2021 were type 2 diabetes (T2D), defined by elevated blood glucose due to deficient insulin production and reduced cell response to insulin.^1^ Youth-onset diabetes in previous decades largely referred to type 1 diabetes, but the prevalence of youth-onset T2D has also increased.^2^ About one in five children and adolescents in the United States during 2005–2016 had prediabetes, impaired fasting glucose, impaired glucose tolerance, and/or elevated glycated hemoglobin that would not meet clinical criteria for diabetes but can progress to diabetes over time.^3^ Childhood metabolic health is strongly predictive of adult metabolic disease, and youth-onset T2D is associated with worse outcomes and complications.^2,4^ Mexico has among the highest diabetes and youth-onset T2D rates in the world.^5^

Metals and metalloids (henceforth, metals) have been linked to T2D, impaired fasting glucose, and insulin resistance, although prospective studies among children and adolescents are limited. Nonessential metals arsenic (As), lead (Pb), and cadmium (Cd) are first, second, and seventh on the US Agency for Toxic Substances and Disease Registry’s Toxic Substance Priority List^6^ and have all been connected with T2D risk or outcomes in epidemiological studies in adults,^7–11^ including in Mexico.^12^ In mouse models and in vitro, As exposure has been shown to cause oxidative stress and damage pancreatic beta cells responsible for insulin production, as well as contribute to general inflammation and disrupted insulin signaling.^8^ There is also toxicological evidence that Cd can disrupt pancreatic function and increase diabetes risk.^13^

Essential metals have also been associated with T2D risk. Essential metals are critical for physiological function within a given range and can be harmful at both deficient and excessive levels.^14^ Both selenium (Se) and zinc (Zn) are incorporated into a wide range of functional proteins, and the abundant selenoprotein P (SeP) has been connected to insulin resistance.^15^ In mouse models, high-Se diets have been connected to insulin resistance.^16^ Experimental evidence also demonstrates that cobalt (Co) and Zn are involved in insulin synthesis, activity, and storage, and that Se and manganese (Mn) have antioxidant properties, which may mitigate oxidative damage related to impaired insulin production or signaling.^17^ Selenium and Zn supplementation for preventing and managing diabetes have been studied in clinical trials, but results have been mixed.^18–20^ There is epidemiological evidence of an association between high Se exposure and slightly increased T2D risk^21^ and between Mn and higher fasting plasma glucose (FPG) and insulin resistance.^22^ Epidemiological evidence has documented decreased blood Zn among people with T2D.^23^ Most studies have been conducted in adults, although cross-sectional studies in children have found associations between urinary metal mixtures and metabolic parameters.^24,25^ Early life may be a particularly vulnerable period for environmental exposures, including metals.^26^ Metabolic dysregulation in early life may lead to increased risk of lifelong metabolic disease, highlighting the importance of characterizing these associations in children.^27,28^

Most previous work has evaluated the effects of individual metals, but metal exposure occurs in complex mixtures. Thus, models only accounting for individual metals may overlook important joint impacts or mis-specify individual associations. We aimed to evaluate individual and joint associations between early childhood metal exposures in whole blood at age 4 and glucose and insulin homeostasis across four visits from approximately 4 to 12 years of age.

## Methods

### Study design and population

We used data from the Programming Research in Obesity, Growth, Environment, and Social Stressors (PROGRESS) prospective birth cohort study. The PROGRESS study recruited 1054 pregnant women between 2007 and 2011 through the Mexican Social Security System (Instituto Mexicano del Seguro Social), 948 of whom continued follow-up after delivery.^29,30^ To be eligible, women had to be <20 weeks gestation, ≥18 years old, free of heart or kidney disease, have access to a telephone, plan to reside in Mexico City for the next 3 years, not use steroids (including glucocorticoids) or anti-epilepsy drugs, and not consume alcohol on a daily basis.^29^ At the 4-year visit, 500 children provided blood for metal measurements. At the age 4-, 6-, 8-, and 10-year visits, 434 children provided blood for at least one measure of FPG, insulin, or homeostatic model assessment of insulin resistance (HOMA-IR) and β-cell function (HOMA-β). We excluded six children with FPG values above the American Diabetes Association cutoff for diabetes diagnosis, five missing environmental tobacco smoke (ETS) exposure information, and one reporting maternal smoking during pregnancy. Our study population consists of 422 children with complete data on blood metals, covariates, and all outcomes for at least one follow-up visit (Figure S1, https://links.lww.com/EE/A487). Characteristics of mother–child dyads included in the study population (n = 422) were similar to those in the PROGRESS cohort overall (n = 948, Table S1, https://links.lww.com/EE/A487).

The institutional review boards of participating institutions approved all study protocols (Instituto Nacional de Salud Publica de Mexico project #560, National Institute of Perinatology (Mexico) 2024-1-12, Icahn School of Medicine Human Subjects Management #12-00751, and Columbia University Human Research Protection Office protocol #AAAR0689).

### Measurement of blood metals

Blood samples from the 4-year visit were collected in metal-free tubes and refrigerated at 4°C until shipment to the Trace Metals Laboratory at the Icahn School of Medicine at Mount Sinai, New York, NY, where they were frozen at −20°C until analysis. Blood elemental concentrations have been shown to be stable at ≤4°C.^31^ After digestion in concentrated HNO_3_ and 30% H_2_O_2_, As, Cd, Cs, Co, Cr, Cu, Mn, Pb, Sb, Se, and Zn were measured in whole blood using the Agilent 8800 ICP Triple Quad (Agilent, Santa Clara, CA) in tandem mass spectrometry mode, with quality controls as previously described for Pb.^32^ Limits of detection (LODs) and number of samples below the LOD are provided in Table 2. Values below LODs are often censored in lab outputs or data analyses, but because values below LODs were available, we used these in further analyses instead of imputed values.^29,33^

### Measurement of metabolic outcomes

At the 4-, 6-, 8-, and 10-year visits, blood samples were analyzed for plasma glucose (mg/dL) using the respons910 automated analyzer (DiaSys Diagnostic Systems, Holzheim, Germany). Before the 4-year visit, children were not asked to fast due to their young age. Beginning at the 6-year visit, children were asked to fast overnight before the visit. At the 8- and 10-year visits, blood samples were additionally analyzed for fasting serum insulin (µU/mL) using the Immulite 1000 analyzer (Siemens, Germany) through March 2020 (8-year visit: all participants, n = 306; 10-year visit: n = 38) and the Architect i1000 (Abbott Laboratories, Abbott Park, IL) after June 2020 (10-year visit: n = 362). Among participants at the 10-year visit, insulin did not differ by instrument (*P* value associated with Wilcoxon rank sum for difference = 0.11). We used the HOMA framework^34^ to estimate insulin resistance (HOMA-IR) and β-cell function (HOMA-β) using the following equations:


$$\begin{array}{c} HOMA\text{-}IR=\frac{Fasting\;glucose\;\left(\frac{mg}{dL}\right)\times fasting\;insulin\left(\frac{\mu U}{mL}\right)}{405} \end{array}$$

$$\begin{array}{c} HOMA\text{-}\beta =\frac{Fasting\;insulin\left(\frac{\mu U}{mL}\right)\times 360}{Fasting\;glucose\left(\frac{mg}{dL}\right)-63} \end{array}$$

Six observations with negative HOMA-β values (fasting glucose <63 mg/dL) were excluded from analyses.

### Covariates

We identified sex, maternal education (<high school, high school, or >high school), maternal socioeconomic status (SES), and exposure to ETS (i.e., smoker(s) in the household vs. none) as factors likely to influence both blood metals and metabolic outcomes (FPG, insulin, HOMA-IR, and HOMA-β) (Figure S2, https://links.lww.com/EE/A487). We also included age (continuous) and season (cold/dry: November–February, warm/dry: March–April, or rainy: May–October) at outcome as precision covariates. While body mass index-for-age *z*-score (*z*BMI) and puberty status could be related to both metals and outcomes, we included these only in sensitivity analyses because we hypothesized they might be on the causal pathway from exposure to outcomes. We also conducted sensitivity analyses additionally adjusting for child diet (seafood, fast food, fruit and vegetable [continuous, g/d], and total energy [continuous, kilocalories/d] intake) and maternal characteristics (age [continuous], parity [first birth vs. not]).

Covariates were obtained at enrollment (maternal education, SES, age, and parity), at birth (child sex), at the 4-year visit (ETS, diet), and at outcome measurement (age, season, *z*BMI, and puberty status). At each visit, study staff administered validated questionnaires to collect demographic, lifestyle, and environmental information. Maternal SES was estimated using the Mexican Association of Market and Public Opinion Research Agencies index, which classifies households into six categories based on 13 factors (e.g., head-of-household education, home appliances, car ownership).^35^ We further grouped these into lower, medium, and higher SES relative to our study population.

Study staff measured child height and weight using a professional digital scale (Health-o-meter), and we calculated zBMI using World Health Organization guidelines.^36^ Puberty was assessed at the 8- and 10-year visits using Tanner staging. Trained pediatricians evaluated genital/testicular development for boys (using an orchidometer) and breast development for girls, assigning stages from 1 (prepubertal) to 5 (full maturity).^37,38^ For some participants, pediatrician evaluations were not available, and we used participants’ self-report (e.g., “Choose the drawing closest to your stage of development”). We categorized puberty as early (Tanner stage 1–2), mid (3), or late (4–5). Seafood, fast food, fruit and vegetable, and total energy intake were estimated from a 140-item validated food frequency questionnaire administered to mothers to assess children’s dietary patterns over the past week.^39^

### Statistical analyses

Because blood metal distributions were right-skewed and linear regression is sensitive to extreme values, we log_2_-transformed blood metal concentrations. We evaluated correlations between each pair of metals using Spearman’s rank correlation coefficients. Insulin, HOMA-IR, and HOMA-β values were also right-skewed, so we log_2_-transformed these values as well. We evaluated correlations between each pair of outcomes using Spearman’s rank correlation coefficients and stability over time using intraclass correlation coefficients.

We used generalized additive mixed models to evaluate if relationships between metals and metabolic outcomes departed from linearity. We used the R package *mgcv*^40^ to fit models for each metal and outcome, fitting the effect of that metal as a smooth function using restricted maximum likelihood (REML) to select smoothing parameters, adjusting for covariates, and with a random intercept for each participant. Because no associations between metals and outcomes with *P* < 0.05 had effective degrees of freedom (reflecting degree of nonlinearity) greater than 1 (Table S3, https://links.lww.com/EE/A487), we modeled metals linearly in further models.

We estimated associations of individual blood metals with metabolic outcomes using linear mixed effects models. We fit separate models for each outcome, adjusted for covariates and other metals, with a random intercept for each participant. All variance inflation factors for metals were less than 2, indicating low multicollinearity. For generalized additive mixed models and linear mixed effects models, we report effect estimates and 95% confidence intervals (CIs), as well as both unadjusted and Bonferroni-adjusted *P* values (to account for testing 11 metals within each outcome), but interpret findings primarily based on effect estimates and precision.

To evaluate joint associations of metal mixtures with metabolic outcomes, we used quantile g-computation. We used the R package *qgcomp*^41^ to fit separate models for each outcome, each as both non-bootstrapped and bootstrapped with 5000 iterations, adjusting for covariates and other blood metals and accounting for clustering of observations by participant using cluster-based bootstrapping.^42^ We report the mixture effect, Ψ, of a one-quartile increase in all components of the metal mixture on each outcome (bootstrapped) and the relative contribution (weight, non-bootstrapped) of each individual metal to effects in either positive or negative directions.

All statistical analyses were conducted in R version 4.5.1.^44^

### Secondary analyses

Effects of metals can differ by sex; for example, differences in iron status can affect metal absorption, and estrogen can stimulate the production of metallothioneins and other metal-binding proteins.^43^ Therefore, we also ran separate linear mixed effects and quantile g-computation models for male and female children. We tested for effect modification by running additional models with interaction terms for each metal and sex, using the R package *qgcompint*^45^ for quantile g-computation models.

We ran linear mixed effects and quantile g-computation analyses, additionally adjusting for (1) *z*BMI, (2) puberty status, (3) child diet, and (4) maternal characteristics. We also ran linear mixed effects and quantile g-computation analyses excluding age 4-year FPG measures (because we were unsure of children’s fasting status). We also ran sensitivity analyses excluding log_2_-transformed metals more than 2.5 standard deviations from the mean.

## Results

### Study population characteristics

The study population included 422 children with blood metals measured at the age 4-year visit and at least one visit with complete covariate and outcome data for each outcome (Figure S1, https://links.lww.com/EE/A487). Because not all outcomes were measured at all visits—insulin, HOMA-IR, and HOMA-β were only assessed at the 8- and 10-year visits—the number of available observations differed by outcome (N = 1504 visits for FPG, 706 for insulin, 704 for HOMA-IR, and 698 for HOMA-β).

Children’s characteristics were similar across the four follow-up visits (Table 1). Approximately half of children were male. Most mothers had lower socioeconomic status (54%–58%) and a high school education or less. Few (13%–14%) reported any smoker(s) in the home at the age 4-year visit.

**Table 1.** Participant characteristics by visit (N = 422)

		At least one outcome measured at…			
Characteristic	Metals measured at age 4-year visit, N = 422^*a*^	4-year visit, N = 377^*a*^	6-year visit, N = 328^*a*^	8-year visit, N = 401^*a*^	10-year visit N = 400^*a*^
Baseline					
Sex					
Female	217 (51%)	191 (51%)	164 (50%)	208 (52%)	208 (52%)
Male	205 (49%)	186 (49%)	164 (50%)	193 (48%)	192 (48%)
SES					
Low	229 (54%)	208 (55%)	189 (58%)	220 (55%)	217 (54%)
Medium	153 (36%)	134 (36%)	105 (32%)	144 (36%)	146 (37%)
High	40 (9.5%)	35 (9.3%)	34 (10%)	37 (9.2%)	37 (9.3%)
Maternal education					
<High school	173 (41%)	154 (41%)	140 (43%)	166 (41%)	162 (41%)
High school	158 (37%)	141 (37%)	116 (35%)	150 (37%)	151 (38%)
>High school	91 (22%)	82 (22%)	72 (22%)	85 (21%)	87 (22%)
ETS	56 (13%)	50 (13%)	45 (14%)	52 (13%)	54 (14%)
Time-varying					
Age	4.75 (4.35, 5.10)	4.77 (4.36, 5.10)	6.62 (6.31, 6.98)	9.65 (9.27, 10.25)	12.83 (12.21, 13.43)
Date	2013-01-03 to 2015-10-08	2013-01-03 to 2015-10-08	2014-10-06 to 2017-03-24	2018-04-25 to 2020-01-31	2020-01-13 to 2023-08-08
Season					
Cold/dry	120 (28%)	100 (27%)	126 (38%)	84 (21%)	158 (40%)
Warm/dry	86 (20%)	81 (21%)	60 (18%)	31 (7.7%)	67 (17%)
Rainy	216 (51%)	196 (52%)	142 (43%)	286 (71%)	175 (44%)
zBMI	0.12 (−0.50, 0.70)	0.14 (−0.47, 0.70)	0.13 (−0.57, 0.93)	0.79 (−0.08, 1.94)	0.79 (−0.16, 1.73)
Unknown	0	0	0	2	0
Puberty status					
Early (Tanner 1–2)	-	-	-	330 (86%)	88 (22%)
Mid (Tanner 3)	-	-	-	10 (2.6%)	131 (33%)
Late (Tanner 4–5)	-	-	-	43 (11%)	179 (45%)
Unknown	-	-	-	18	2
FPG (mg/dL)	86 (79, 92)	86 (79, 92)	86 (81, 92)	86 (79, 92)	86 (80, 92)
Unknown	45	0	0	2	0
IFG	37 (9.8%)	37 (9.8%)	16 (4.9%)	27 (6.8%)	26 (6.5%)
Unknown	45	0	0	2	0
Serum insulin (µU/mL)	-	-	-	8 (5, 14)	11 (8, 16)
Unknown	-	-	-	95	0
HOMA-IR	-	-	-	1.76 (0.92, 2.98)	2.37 (1.72, 3.34)
Unknown	-	-	-	97	0
HOMA-β	-	-	-	139 (82, 253)	196 (128, 293)
Unknown	-	-	-	99	4

^*a*^ N (%), median (IQR), range.

IQR indicates interquartile range; SES, socioeconomic status; ETS, exposure to environmental tobacco smoke defined by any smoker(s) in home at 4-year visit; *z*BMI, BMI-for-age (*z*-score); FPG, fasting plasma glucose; IFG, impaired fasting glucose (100–125 mg/dL); HOMA-IR, homeostatic model assessment of insulin resistance; HOMA-β, homeostatic model assessment of β-cell function..

Children’s median age at the 4-year visit was 4.8 years (interquartile range [IQR] 4.4, 5.1); at the 6-year visit, 6.6 (IQR 6.3, 7.0); because of scheduling delays and the coronavirus disease 2019 (COVID-19) pandemic, at the 8-year visit, 9.7 (IQR 9.3, 10.3); and at the 10-year visit 12.8 (IQR 12.2, 13.4). The 4- and 6-year visits were conducted from 2013 to 2017, and the 8- and 10-year visits were conducted from 2018 to 2023. Median *z*BMI was near zero at the 4- and 6-year visits but increased to approximately 0.8 at the 8- and 10-year visits. At the 8-year visit, most children were in early puberty (Tanner stages 1–2; 86%), while by the 10-year visit, most had progressed to mid or late puberty (Tanner stages 3–5; 78%).

Median FPG was stable across all visits at 86 mg/dL. The prevalence of impaired fasting glucose (IFG; ≥100 mg/dL)^46^ was 4.9% at the 6-year visit, 6.8% at the 8-year visit, and 6.5% at the 10-year visit. Fasting serum insulin, HOMA-IR, and HOMA-β were only assessed at the 8- and 10-year visits; median insulin increased from 8 µU/mL (IQR: 5, 14) at the 8-year visit to 11 µU/mL (IQR: 8, 16) at the 10-year visit, while median HOMA-IR increased from 1.76 (IQR: 0.92, 2.98) to 2.37 (IQR: 1.72, 3.34), and median HOMA-β increased from 139 (IQR: 82, 253) to 196 (IQR: 128, 293).

For most blood metals (As, Cs, Pb, Sb, Co, Cu, Mn, Se, and Zn), no children’s measurements were below the instrument LOD. For Cr, 11% of measurements were below the LOD; for Cd, 3.1% were below the LOD (Table 2). The strongest correlations between metals were between Sb and Co (*ρ* = 0.58), Cu and Zn (*ρ* = 0.57), Se and Zn (*ρ* = 0.54), and Cr and Sb (*ρ* = 0.52) (Figure S3, https://links.lww.com/EE/A487).

**Table 2.** Blood metal concentrations (µg/L) at age 4-year visit (N = 422)

Metal	Median (IQR)	Range	LOD range	n (%) below LOD
As	0.45 (0.36, 0.6)	0.17–11	0.023–0.086	0 (0%)
Cd	0.074 (0.055, 0.1)	0.014–0.39	0.0097–0.062	13 (3.1%)
Cr	0.49 (0.38, 0.61)	0.13–2.8	0.098–1	47 (11%)
Cs	2.3 (1.9, 2.7)	0.88–5.2	0.016–0.079	0 (0%)
Pb	17 (13, 25)	5.2–230	0.017–0.13	0 (0%)
Sb	4.9 (4.3, 5.7)	3–26	0.024–0.27	0 (0%)
Co	0.16 (0.14, 0.19)	0.084–1.2	0.015–0.077	0 (0%)
Cu	970 (840, 1100)	450–1700	0.23–1.5	0 (0%)
Mn	11 (8.9, 13)	3.6–25	0.083–0.39	0 (0%)
Se	190 (180, 210)	99–280	0.2–4.4	0 (0%)
Zn	4900 (4300, 5700)	3000–12,000	1.4–7.4	0 (0%)

### Individual associations of metals and metabolic health

Each doubling of blood Se concentration at the 4-year visit was associated with an average 5.8 mg/dL increase in FPG across 4-, 6-, 8-, and 10-year visits (95% confidence interval [CI] 1.9, 9.7) (Figure 1, Table S5, https://links.lww.com/EE/A487). Blood Se was also associated with higher average insulin and HOMA-IR across 8- and 10-year visits (adjusted geometric mean ratio [aGMR] 1.6 [95% CI 1.1, 2.3] and aGMR 1.6 [95% CI = 1.1, 2.4], respectively, Table S6, https://links.lww.com/EE/A487). Blood Zn was associated with lower average insulin and HOMA-IR (aGMR 0.75 [95% CI = 0.58, 0.98] and aGMR 0.74 [95% CI = 0.57, 0.98], respectively). Only the association of Se with average FPG was robust to Bonferroni correction; associations of Se with insulin and HOMA-IR and negative associations of Zn with insulin and HOMA-IR were not robust to correction (Table S4, https://links.lww.com/EE/A487). No metals were significantly associated with HOMA-β.

**Figure 1.**
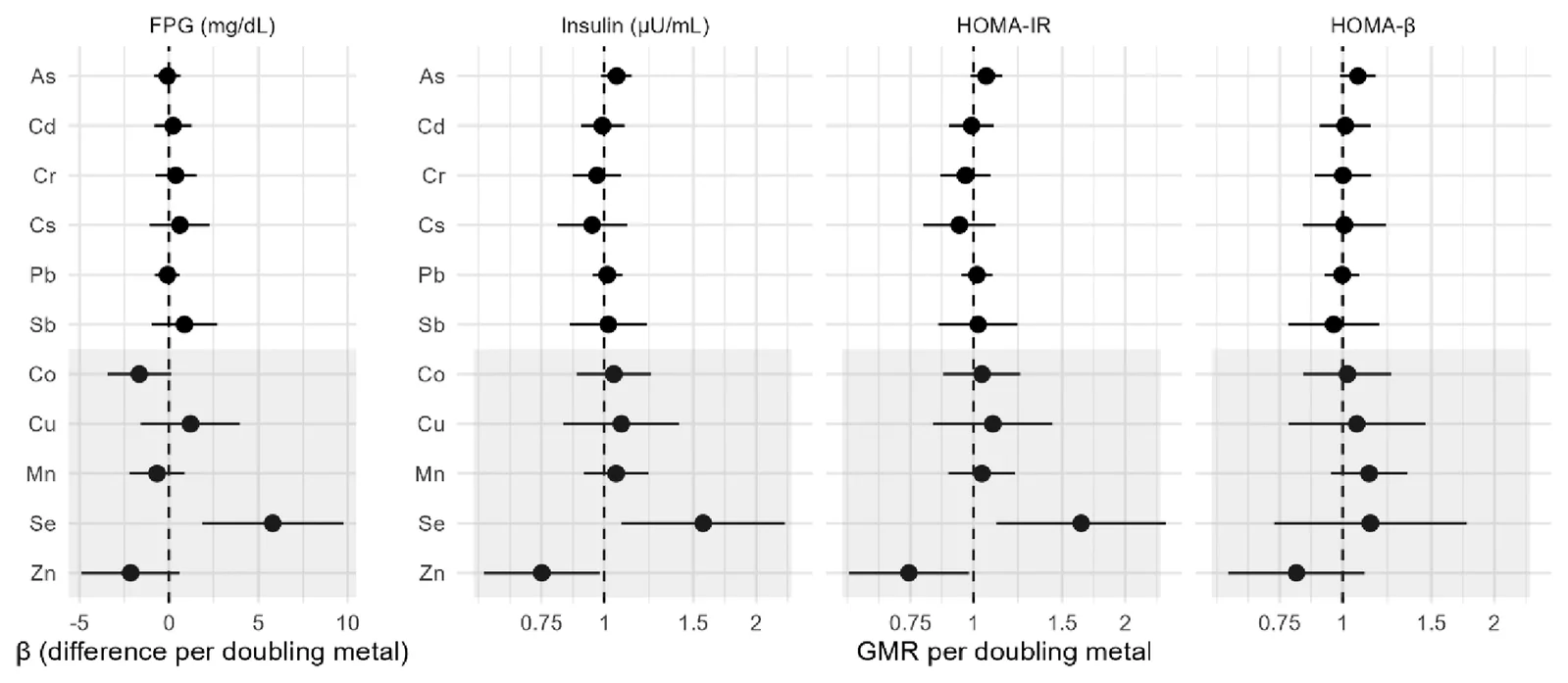
Associations of blood metal concentrations (age 4-year visit) with average metabolic outcomes at 4-, 6-, 8-, and 10-year visits (N = 422). All models adjusted for sex, maternal education, maternal socioeconomic status, exposure to environmental tobacco smoke defined by any smoker(s) in home at 4-year visit, age and season at outcome, and all other metals. Circles represent point estimates; lines 95% confidence intervals. Insulin, HOMA-IR, and HOMA-β were log_2_-transformed, so associations were exponentiated to geometric mean ratios (GMRs). All models include the same 422 participants (1504 observations for FPG, 706 for insulin, 704 for HOMA-IR, and 698 for HOMA-β).

### Joint associations of metals and metabolic health

Blood metal concentrations at the 4-year visit were jointly associated with average insulin and HOMA-IR at the 8- and 10-year visits (Table 3). A simultaneous quartile increase in all log_2_-transformed metals was associated with a 1.10-fold increase in insulin (95% CI = 1.01, 1.21) and 1.11-fold increase in HOMA-IR (95% CI = 1.01, 1.22). The joint association with HOMA-β was in the same direction, but the 95% CI included the null (aGMR 1.08 [95% CI = 0.96, 1.21]), and there was no meaningful joint association with FPG (adjusted change 0.93 [95% CI = −0.084, 1.9 mg/dL]). Arsenic and Se had the largest positive weights (0.29 and 0.27, respectively, for insulin; 0.29 and 0.29 for HOMA-IR), indicating they were the primary drivers of the adverse mixture effect. Zn had the largest negative weight for both outcomes (0.58).

**Table 3.** Quantile g-computation estimates (joint associations and 95% confidence intervals; weights) for simultaneous quartile increase in all log_2_-transformed age 4-year visit blood metal concentrations (N = 422)

Outcome (age 4–10 visits)	Joint association per quartile increase	Metal	Negative weight	Positive weight
FPG (mg/dL)	Adjusted change 0.93 (−0.084, 1.9)			
		Se	-	0.47
		As	-	0.18
		Cu	-	0.13
		Cs	-	0.076
		Sb	-	0.074
		Mn	-	0.035
		Cr	-	0.023
		Pb	-	0.017
		Co	0.49	-
		Zn	0.45	-
		Cd	0.06	-
Serum insulin (µU/mL)	**GMR 1.10 (1.01, 1.21**)			
		As	-	0.29
		Se	-	0.27
		Cu	-	0.26
		Pb	-	0.071
		Mn	-	0.06
		Co	-	0.055
		Zn	0.58	-
		Cd	0.19	-
		Cs	0.12	-
		Cr	0.088	-
		Sb	0.021	-
HOMA-IR	**GMR 1.11 (1.01, 1.22**)			
		Se	-	0.29
		As	-	0.29
		Cu	-	0.24
		Pb	-	0.11
		Mn	-	0.04
		Co	-	0.026
		Sb	-	0.005
		Zn	0.58	-
		Cd	0.19	-
		Cs	0.16	-
		Cr	0.07	-
HOMA-β	GMR 1.08 (0.96, 1.21)			
		Cu	-	0.35
		As	-	0.22
		Mn	-	0.11
		Cr	-	0.076
		Cs	-	0.074
		Cd	-	0.071
		Se	-	0.052
		Co	-	0.05
		Zn	0.54	-
		Sb	0.29	-
		Pb	0.17	-

All models adjusted for sex, maternal education, maternal socioeconomic status, exposure to environmental tobacco smoke defined by any smoker(s) in home at 4-year visit, age and season at outcome, and all other metals. Serum insulin, HOMA-IR, and HOMA-β were log_2_-transformed, so joint associations were exponentiated to geometric mean ratios (GMRs). Weights represent the proportion of the positive or negative partial effect for each component (metal) in the mixture on metabolic outcomes; negative weights and positive weights each sum to 1. All models include the same 422 participants (1504 observations for FPG, 706 for insulin, 704 for HOMA-IR, and 698 for HOMA-β).

### Secondary analyses

In sex-stratified linear mixed effects models, associations of Se with average FPG, as well as negative associations of Zn with insulin and HOMA-IR, were somewhat stronger among female versus male participants (Figure S5, https://links.lww.com/EE/A487). Among female participants only, As and Cu were associated with higher insulin and HOMA-IR. However, the *P* value for interaction between each metal and sex was >0.05 for all metals and outcomes.

In sex-stratified quantile g-computation models, there was a joint association between all metals and FPG among male participants only (average increase of 1.7 mg/dL FPG at age 4- to 10-year visits per quartile increase in all log_2_-transformed metals, 95% CI = 0.29, 3.2, Table S7, https://links.lww.com/EE/A487). Relative weights of individual metals also differed by sex: As had the highest positive weight (0.31) among male participants, while Se had the highest positive weight (0.76) among female participants. However, the *P* value for interaction between metals and sex was >0.05, and there was no evidence of interaction between metals jointly and sex for insulin, HOMA-IR, or HOMA-β (Table S8, https://links.lww.com/EE/A487).

Associations between Se and average FPG were robust to excluding age 4-year FPG. A doubling in blood Se at the 4-year visit was associated with an average increase of 6.1 mg/dL FPG at the 6- to 10-year visits (95% CI = 1.9, 10), adjusted for covariates and other metals (Table S5, https://links.lww.com/EE/A487). Excluding age 4-year FPG did not change the null joint association of all blood metals (Table S9, https://links.lww.com/EE/A487).

The positive association of Se with FPG was robust to additional adjustment for zBMI, diet, and maternal age and parity, but not puberty status at outcome, while the associations of Se with insulin and HOMA-IR were robust to additional adjustment for puberty, diet, and maternal age and parity, but not zBMI (Tables S5 and S6, https://links.lww.com/EE/A487). The negative association of Zn with insulin and HOMA-IR was robust to adjustment for diet and maternal age and parity, but not to zBMI or puberty (Table S6, https://links.lww.com/EE/A487). In contrast, positive joint associations of all blood metals with HOMA-IR were robust to all additional adjustments (Table S10, https://links.lww.com/EE/A487), though joint associations with insulin were robust only to adjustment for zBMI, not puberty, diet, or maternal age and parity. Excluding log_2_-transformed metals more than 2.5 standard deviations from the mean (n = 16 observations from four participants for Mn to 64 observations from 16 participants for Sb) did not qualitatively change individual-metal results (not shown).

## Discussion

Among the study population of 422 children in Mexico City, blood metal levels at age 4 were jointly associated with higher average insulin and HOMA-IR at 8- and 10-year visits. Selenium was associated with higher FPG, insulin, and HOMA-IR at up to four visits from 4 to 10 years, while Zn was negatively associated with insulin and HOMA-IR.

### Selenium

The positive association of blood Se with FPG, insulin, and HOMA-IR is consistent with a growing body of evidence suggesting Se may impair glucose homeostasis at elevated concentrations. Se is an essential micronutrient that can be hazardous at high levels.^47^ Participants’ whole blood Se (median 190 µg/L) was comparable to US adult general population levels (weighted median 192 µg/L; 2011–2020), where higher blood Se has also been associated with increased diabetes risk.^48^ Excess Se may increase oxidative stress and has been shown to induce insulin resistance in animal models.^47,49^ In a Mendelian randomization study, Cheng et al^50^ found that genetically predicted blood selenium was associated with increased risk of T2D in adults, partially mediated by increased BMI. Prior evidence in children is limited: one study found no association between dietary Se and elevated FPG in Brazilian adolescents,^51^ although blood Se levels have been linked with obesity among Polish children.^52^

Our findings add longitudinal evidence that blood Se in early childhood may be prospectively associated with glycemic outcomes, although modest effect estimates and attenuation after adjusting for *z*BMI and pubertal status leave the causal structure uncertain. One possibility is that adiposity and pubertal development partially mediate the relationship between Se and glycemic outcomes. Alternatively, *z*BMI and pubertal status may act as confounders if children with different growth trajectories have differing Se exposure for reasons unrelated to the causal pathway. Additionally, we evaluated 11 metals, and only the Se-FPG association remained significant after Bonferroni correction; Se-insulin and Se-HOMA-IR associations were not robust to Bonferroni correction. Nonetheless, Se associations were directionally consistent across glycemic outcomes and stable across adjustment for diet, maternal age, and parity; we interpret these results as hypothesis-generating and in need of replication.

### Zinc

The negative association of Zn with insulin and HOMA-IR is consistent with evidence of Zn dysregulation early in the development of insulin resistance and T2D. Zinc is primarily obtained from diet and drinking water and is essential for insulin synthesis, storage, and activity, but excess free Zn can also exert toxic effects.^53^ Zinc is tightly regulated in blood, so levels represent physiological processes in addition to exposure.^23^ Epidemiological studies have shown lower whole blood Zn after T2D diagnosis.^54^ Some supplementation trials in adults and children have found associations with lower glycemia and insulin resistance,^18,55^ but others have found no effect.^23,56^ Our findings are consistent with lower blood Zn as an early indicator of processes related to insulin resistance or a protective role of Zn on insulin resistance during childhood. As with Se, this association did not persist after adjusting for *z*BMI and pubertal status, and the Zn-HOMA-IR association was not robust to Bonferroni correction, complicating inference.

### Joint mixture effects

We found joint associations of metals with higher insulin and HOMA-IR. The joint association with HOMA-IR persisted after adjusting for *z*BMI and pubertal status, even as individual metal associations were attenuated. Co-occurring metal exposures may contribute to insulin resistance through pathways not fully captured by individual metal analyses, and the cumulative burden of the mixture may be more robustly associated with metabolic health than any single metal. Our findings are consistent with prior cross-sectional studies in children, which found associations between urinary metal mixtures and FPG^24^ and HOMA-IR.^25^ Our findings contrast with a prior study of prenatal metal mixtures and early childhood cardiometabolic health in the same cohort, which found associations of the overall metal mixture with lower HbA1c, a marker of average blood sugar over 2 to 3 months, driven largely by essential metals.^33^ This difference could be due to developmental timing—essential metal deficiencies may be more detrimental prenatally versus during early childhood. Our findings also contrast with a closely related preprint, in which we did not find associations of early childhood blood metals with FPG (individually or as a mixture) or with HOMA-IR (as a mixture).^57^ We believe the current analysis is more robust and reliable: we used repeated outcome measurements and serum instead of plasma insulin, after learning about an instrument limitation related to plasma insulin measurements before June 2020.

Within the mixture, As contributed the largest positive weight alongside Se, despite no significant individual association with metabolic outcomes. Blood As levels (median 0.45 µg/L) were similar to an adolescent cohort in Uruguay (median 0.37 µg/L),^58^ and lower compared with a general population cohort in Germany (median 0.8 µg/L).^59^ Arsenic has been shown to affect beta cells and glucose homeostasis in rodents.^60^ In a Mexican adult cohort, higher As was associated with higher body weight and higher FPG.^12^ Systematic reviews of epidemiological studies have found consistent associations between As and T2D in adults.^61^ Arsenic exposure has been associated cross-sectionally with insulin resistance in adolescents.^62^ Our findings add to evidence that As, in combination with other metal exposures, could affect insulin resistance in children and adolescents.

### Sex differences

We found no evidence of significant effect modification by sex. However, in sex-stratified models, we observed a pattern of stronger associations among female compared with male participants for several individual metals, including associations of As and Cu with insulin and HOMA-IR among female participants only. These patterns could reflect sex differences in exposure or metal metabolism. A study in Bangladesh found stronger associations of As with elevated FPG and T2D risk among women versus men.^63^ However, women have been shown to generally have better As methylation capacity than men, allowing for faster clearance of inorganic As from the body, and thus potentially lower toxicity.^64^ Yu et al^25^ also found sex differences in associations of metals with FPG, but found associations of As with FPG and Zn with HOMA-IR among boys but not girls.

Our sex-stratified findings should be interpreted cautiously as exploratory. Larger studies with adequate power to formally test sex interactions are needed to determine whether sex modifies the relationship between metal exposures and glycemic health in children and adolescents.

### Strengths and limitations

This study has several strengths. The longitudinal design with up to four follow-up visits allowed us to estimate associations of metals with average glycemic outcomes across childhood and early adolescence, reducing the influence of visit-specific measurement variability and allowing us to assess exposure before outcomes. The use of quantile g-computation enabled simultaneous estimation of mixture effects and the relative contribution of individual metals, providing a more realistic characterization of real-world co-exposures than single-pollutant approaches.^41^ The panel of eleven metals captured a broad range of potentially relevant exposures, and the cohort’s well-characterized socioeconomic and demographic profile supported adjustment for key confounders.

Several limitations should be noted. Metal levels in whole blood provide an estimate of exposure from many sources and exposure routes (e.g., ingestion, inhalation, dermal). However, metabolic biotransformation and elimination affect how accurately blood metal biomarkers represent body burden as well as the exposure window over which biomarkers represent body burden.^65^ For example, the preferred chronic exposure estimates are generally urine for As and bone for Pb.^65^ Also, blood Cu and Zn are tightly physiologically regulated and may reflect alterations in metal homeostasis rather than exposure.^65^ Repeated metal measurements would have better reflected long-term exposure relevant to health effects. Also, the toxicity of Se and As depends on the chemical species,^47,66^ which we did not assess in this analysis. Additionally, some measurements were below the LOD for Cr and Cd; we used instrument-reported measurements, which preserve some information on ranking of measurements but carry meaningful measurement error. This error would be expected to reduce precision for Cr and Cd, both of which were null across models. We also investigated associations between 11 metals and four interrelated metabolic health outcomes; some observed associations may reflect chance findings arising from multiple comparisons.

Plasma is generally preferred for HOMA models, yet only serum insulin was available as a reliable measure for this analysis.^34^ Nonetheless, serum insulin can be used to estimate insulin resistance.^67^ Fasting glucose may also have poorer sensitivity for identifying glucose dyshomeostasis in pediatric compared with adult populations.^68^ Additionally, because glucose measures were missing at some visits for some participants, it is possible that some children with diabetes were included in the study. While we adjusted for a range of potential confounders, unmeasured and residual confounding cannot be excluded. Diet affects both metal exposures and metabolic health; while additional adjustment for seafood, fast food, fruit and vegetable, and total energy intake did not qualitatively alter most effect estimates, other aspects of diet could be important for exposures and outcomes. We also used a food frequency questionnaire querying intake during the previous week, which may not capture long-term patterns. While we sought to account for individual-level socioeconomic status, we did not account for neighborhood-level deprivation, food environment, housing characteristics relevant to metal exposures, or other environmental co-exposures. The attenuation of individual metal associations after adjusting for *z*BMI and pubertal status raises unresolved questions about the causal structure of these relationships that the current study design cannot address. Our findings also may not be generalizable to populations with differing dietary and environmental metal exposure levels.

## Conclusions

In this longitudinal study of Mexico City children followed from approximately age 4–12 years, early-life blood Se was prospectively associated with higher average FPG, insulin, and HOMA-IR, while blood Zn was associated with lower insulin and HOMA-IR. The metal mixture was jointly associated with higher insulin and HOMA-IR, driven primarily by Se and As. All individual metal-outcome associations and the joint metal-HOMA-IR association were robust to additional adjustment for dietary intake and maternal characteristics. While the individual-metal associations and joint metal mixture-insulin association were attenuated by additional adjustment for BMI or puberty status at outcome measurement, the positive association between the metal mixture and HOMA-IR was not, highlighting the value of mixture modeling for capturing cumulative and potentially synergistic effects. Among the individual–metal associations, only that between Se and FPG remained statistically significant after Bonferroni correction; the remaining individual-metal findings, including sex-stratified associations, should therefore be regarded as hypothesis-generating. We observed a pattern of stronger associations among girls, including associations of As and Cu with insulin and HOMA-IR among girls only. Our findings add to prior evidence from adult and pediatric populations on the importance of early-life metal exposures as potential contributors to metabolic risk. However, because blood concentrations of essential metals such as Se and Zn are influenced by physiological regulation as well as exposure, additional research is needed to clarify whether these associations reflect risks from external exposure, internal processes, or both. Further research should also evaluate adiposity and pubertal development as potential confounders and/or mediators, rigorously examine sex differences, evaluate chemical-chemical interactions, and incorporate other modifiable risk factors.

## ACKNOWLEDGMENTS

We thank all PROGRESS study staff and participants, without whom this research would not be possible.

## Supplementary Material

## References

[1] Ong KL Stafford LK McLaughlin SA. Global, regional, and national burden of diabetes from 1990 to 2021, with projections of prevalence to 2050: a systematic analysis for the Global Burden of Disease Study 2021. Lancet. 2023;402:203–234. 10.1016/S0140-6736(23)01301-6 PMC10364581 37356446

[2] Perng W Conway R Mayer-Davis E Dabelea D. Youth-onset type 2 diabetes: the epidemiology of an awakening epidemic. Diabetes Care. 2023;46:490–499. 10.2337/dci22-0046 PMC10090267 36812420

[3] Andes LJ Cheng YJ Rolka DB Gregg EW Imperatore G. Prevalence of prediabetes among adolescents and young adults in the United States, 2005-2016. JAMA Pediatr. 2020;174:e194498. 10.1001/jamapediatrics.2019.4498 PMC6902249 31790544

[4] Nguyen QM Srinivasan SR Xu JH Chen W Berenson GS. Fasting plasma glucose levels within the normoglycemic range in childhood as a predictor of prediabetes and type 2 diabetes in adulthood: the Bogalusa Heart Study. Arch Pediatr Adolesc Med. 2010;164:124–128. 10.1001/archpediatrics.2009.268 20124140

[5] Dávila-Cervantes CA. The burden of type 2 diabetes in adolescents and young adults in Mexico: analysis from the Global Burden of Disease Study, 1990 to 2019. J Diabetes Metab Disord. 2023;22:1673–1684. 10.1007/s40200-023-01299-x PMC10638125 37975094

[6] ATSDR. Substance Priority List. 2022. Available at: https://www.atsdr.cdc.gov/programs/substance-priority-list.html. Accessed 22 May 2024.

[7] Grau-Perez M Kuo CC Gribble MO. Association of low-moderate arsenic exposure and arsenic metabolism with incident diabetes and insulin resistance in the strong heart family study. Environ Health Perspect. 2017;125:127004. 10.1289/EHP2566 PMC5963590 29373862

[8] Maull EA Ahsan H Edwards J. Evaluation of the association between arsenic and diabetes: a National Toxicology Program workshop review. Environ Health Perspect. 2012;120:1658–1670. 10.1289/ehp.1104579 PMC3548281 22889723

[9] Misra BB Misra A. The chemical exposome of type 2 diabetes mellitus: opportunities and challenges in the omics era. Diabetes Metab Syndr. 2020;14:23–38. 10.1016/j.dsx.2019.12.001 31838434

[10] Planchart A Green A Hoyo C Mattingly CJ. Heavy metal exposure and metabolic syndrome: evidence from human and model system studies. Curr Environ Health Rep. 2018;5:110–124. 10.1007/s40572-018-0182-3 PMC6053628 29460222

[11] Schwartz GG Il’yasova D Ivanova A. Urinary cadmium, impaired fasting glucose, and diabetes in the NHANES III. Diabetes Care. 2003;26:468–470. 10.2337/diacare.26.2.468 12547882

[12] Hernández-Mendoza H Álvarez-Loredo HE Romero-Guzmán ET. Relationship between serum levels of arsenic, cadmium, and mercury and body mass index and fasting plasma glucose in a Mexican adult population. Biol Trace Elem Res. 2022;200:4916–4923. 10.1007/s12011-021-03081-7 PMC8801291 35098445

[13] Chen YW Yang CY Huang CF Hung DZ Leung YM Liu SH. Heavy metals, islet function and diabetes development. Islets. 2009;1:169–176. 10.4161/isl.1.3.9262 21099269

[14] World Health Organization. Principles and Methods for the Assessment of Risk From Essential Trace Elements. 2002. Available at: https://www.who.int/publications/i/item/9241572280. Accessed August 12, 2026.

[15] Misu H Takamura T Takayama H. A liver-derived secretory protein, selenoprotein P, causes insulin resistance. Cell Metab. 2010;12:483–495. 10.1016/j.cmet.2010.09.015 21035759

[16] Zhou J Xu G Bai Z. Selenite exacerbates hepatic insulin resistance in mouse model of type 2 diabetes through oxidative stress-mediated JNK pathway. Toxicol Appl Pharmacol. 2015;289:409–418. 10.1016/j.taap.2015.10.019 26522834

[17] Barra NG Anhê FF Cavallari JF Singh AM Chan DY Schertzer JD. Micronutrients impact the gut microbiota and blood glucose. J Endocrinol. 2021;250:R1–R21. 10.1530/JOE-21-0081 34165440

[18] El Dib R Gameiro OLF Ogata MSP. Zinc supplementation for the prevention of type 2 diabetes mellitus in adults with insulin resistance. Cochrane Database Syst Rev. 2015;2015:CD005525. 10.1002/14651858.CD005525.pub3 PMC9939964 26020622

[19] Stranges S Marshall JR Natarajan R. Effects of long-term selenium supplementation on the incidence of type 2 diabetes: a randomized trial. Ann Intern Med. 2007;147:217–223. 10.7326/0003-4819-147-4-200708210-00175 17620655

[20] Wang X Wu W Zheng W. Zinc supplementation improves glycemic control for diabetes prevention and management: a systematic review and meta-analysis of randomized controlled trials. Am J Clin Nutr. 2019;110:76–90. 10.1093/ajcn/nqz041 31161192

[21] Vinceti M Filippini T Rothman KJ. Selenium exposure and the risk of type 2 diabetes: a systematic review and meta-analysis. Eur J Epidemiol. 2018;33:789–810. 10.1007/s10654-018-0422-8 29974401

[22] Yang J Yang A Cheng N. Sex-specific associations of blood and urinary manganese levels with glucose levels, insulin resistance and kidney function in US adults: national health and nutrition examination survey 2011-2016. Chemosphere. 2020;258:126940. 10.1016/j.chemosphere.2020.126940 32540546

[23] Martinez-Morata I Dennehy K Chepurny O. Type 2 diabetes mellitus and zinc dysregulation: from basic and epidemiological evidence to interventions. J Trace Elem Med Biol. 2025;91:127733. 10.1016/j.jtemb.2025.127733 PMC12703949 40865500

[24] Liu M Guo W Li M. Physical activity modified association of urinary metals mixture and fasting blood glucose in children: from two panel studies. Environ Res. 2024;252:118767. 10.1016/j.envres.2024.118767 38527725

[25] Yu J Yang A Chow E. Environmental exposure to toxic and essential metals and glucose homeostasis in adolescents: a cross-sectional analysis of the Hong Kong School Children Cohort. Ecotoxicol Environ Saf. 2025;305:119179. 10.1016/j.ecoenv.2025.119179 41076865

[26] Haugen AC Schug TT Collman G Heindel JJ. Evolution of DOHaD: the impact of environmental health sciences. J Dev Orig Health Dis. 2015;6:55–64. 10.1017/S2040174414000580 PMC4393755 25471238

[27] Ojanen X Cheng R Törmäkangas T. Towards early risk biomarkers: serum metabolic signature in childhood predicts cardio-metabolic risk in adulthood. EBioMedicine. 2021;72:103611. 10.1016/j.ebiom.2021.103611 PMC8511803 34628356

[28] Morrison JA Glueck CJ Woo JG Wang P. Risk factors for cardiovascular disease and type 2 diabetes retained from childhood to adulthood predict adult outcomes: the Princeton LRC Follow-up Study. Int J Pediatr Endocrinol. 2012;2012:6. 10.1186/1687-9856-2012-6 PMC3466140 22507454

[29] Braun JM Wright RJ Just AC. Relationships between lead biomarkers and diurnal salivary cortisol indices in pregnant women from Mexico City: a cross-sectional study. Environ Health. 2014;13:50. 10.1186/1476-069X-13-50 PMC4068833 24916609

[30] Burris HH Braun JM Byun HM. Association between birth weight and DNA methylation of IGF2, glucocorticoid receptor and repetitive elements LINE-1 and Alu. Epigenomics. 2013;5:271–281. 10.2217/epi.13.24 PMC3787720 23750643

[31] Tevis DS Jarrett JM Jones DR. Assessing the stability of Cd, Mn, Pb, Se, and total Hg in whole human blood by ICP-DRC-MS as a function of temperature and time. Clin Chim Acta. 2018;485:1–6. 10.1016/j.cca.2018.05.043 PMC6544151 29894782

[32] Renzetti S Just AC Burris HH. The association of lead exposure during pregnancy and childhood anthropometry in the Mexican PROGRESS cohort. Environ Res. 2017;152:226–232. 10.1016/j.envres.2016.10.014 PMC5135667 27810680

[33] Kupsco A Kioumourtzoglou MA Just AC. Prenatal metal concentrations and childhood cardio-metabolic risk using Bayesian Kernel machine regression to assess mixture and interaction effects. Epidemiology. 2019;30:263–273. 10.1097/EDE.0000000000000962 PMC6402346 30720588

[34] Matthews DR Hosker JP Rudenski AS Naylor BA Treacher DF Turner RC. Homeostasis model assessment: insulin resistance and beta-cell function from fasting plasma glucose and insulin concentrations in man. Diabetologia. 1985;28:412–419. 10.1007/BF00280883 3899825

[35] Carrasco AV. The AMAI System of Classifying Households by Socio-economic Level. ANA - ESOMAR. 2002. Available at: https://ana.esomar.org/documents/the-amai-system-of-classifying-households-by-socio-economic-level-. Accessed 29 August 2024.

[36] WHO. WHO Child Growth Standards: Length/Height-for-Age, Weight-for-Age, Weight-for-Length, Weight-for-Height and Body Mass Index-for-Age: Methods and Development. 2006. Available at: https://www.who.int/publications/i/item/924154693X. Accessed 29 August 2024.

[37] Marshall WA Tanner JM. Variations in pattern of pubertal changes in girls. Arch Dis Child. 1969;44:291–303. 10.1136/adc.44.235.291 PMC2020314 5785179

[38] Marshall WA Tanner JM. Variations in the pattern of pubertal changes in boys. Arch Dis Child. 1970;45:13–23. 10.1136/adc.45.239.13 PMC2020414 5440182

[39] Denova-Gutiérrez E Ramírez-Silva I Rodríguez-Ramírez S Jiménez-Aguilar A Shamah-Levy T Rivera-Dommarco JA. Validity of a food frequency questionnaire to assess food intake in Mexican adolescent and adult population. Salud Publica Mex. 2016;58:617–628. 10.21149/spm.v58i6.7862 28225938

[40] Wood SN. Generalized Additive Models: An Introduction With R, Second Edition. 2nd ed. Chapman and Hall/CRC; 2017. doi:10.1201/9781315370279

[41] Keil AP Buckley JP O’Brien KM Ferguson KK Zhao S White AJ. A quantile-based g-computation approach to addressing the effects of exposure mixtures. Environ Health Perspect. 2020;128:47004. 10.1289/EHP5838 PMC7228100 32255670

[42] Keil AP. Advanced Topics for the QGcomp Package: Time-to-event, Clustering, Partial Effects, Weighting, Missing Data. 2026. Available at: https://cran.r-project.org/web/packages/qgcomp/vignettes/qgcomp-advanced-vignette.html#ex-cluster. Accessed 12 August 2026.

[43] Lu H Delnicki M Griffin G Wise JL. Current understanding of sex differences in metal-induced diseases. Curr Environ Health Rep. 2025;12:18. 10.1007/s40572-025-00482-x PMC12168244 40202678

[44] *R: The R Project for Statistical Computing*. Available at: https://www.r-project.org/. Accessed 10 December 2025.

[45] Keil A. *qgcompint: Quantile G-Computation Extensions for Effect Measure Modification*. 2025. Available at: https://cran.r-project.org/web/packages/qgcompint/index.html. Accessed 9 April 2025.

[46] American Diabetes Association. Diabetes Diagnosis & Tests. Understanding Diabetes Diagnosis. 2025. Available at: https://diabetes.org/about-diabetes/diagnosis. Accessed 6 May 2024.

[47] ATSDR. Toxicological Profile for Selenium. 2003. Available at: https://www.atsdr.cdc.gov/toxprofiles/tp92.pdf. Accessed 2 January 2025. 38349978

[48] Zhang C Zeng Q Liu X. Association of blood selenium levels with diabetes and heart failure in American general adults: a cross-sectional study of NHANES 2011-2020 pre. Biol Trace Elem Res. 2024;202:3413–3424. 10.1007/s12011-023-03933-4 PMC11144148 37996718

[49] A High-Selenium Diet Induces Insulin Resistance in Gestating Rats and Their Offspring. ScienceDirect. Available at: https://www-sciencedirect-com.ezproxy.cul.columbia.edu/science/article/pii/S0891584912000615. Accessed 19 March 2026. 10.1016/j.freeradbiomed.2012.01.017 PMC3505681 22342560

[50] Cheng L Zeng Z Quan H. Association of selenium with type 2 diabetes and obesity: a univariate and multivariate Mendelian randomization study. Medicine (Baltim). 2025;104:e45338. 10.1097/MD.0000000000045338 PMC12537198 41189202

[51] Retondario A Souza AM Bricarello LP. Selenium intake is not associated with the metabolic syndrome in Brazilian adolescents: an analysis of the study of cardiovascular risk factors in adolescents. Br J Nutr. 2022;127:1404–1414. 10.1017/S0007114521002385 34176526

[52] Blazewicz A Klatka M Astel A. Serum and urinary selenium levels in obese children: a cross-sectional study. J Trace Elem Med Biol. 2015;29:116–122. 10.1016/j.jtemb.2014.07.016 25194955

[53] ATSDR. Toxicological Profile for Zinc. 2005. Available at: https://www.atsdr.cdc.gov/toxprofiles/tp60.pdf. Accessed 12 October 2026. 38377264

[54] Fernández-Cao JC Warthon-Medina M Hall Moran V Arija V Doepking C Lowe NM. Dietary zinc intake and whole blood zinc concentration in subjects with type 2 diabetes versus healthy subjects: a systematic review, meta-analysis and meta-regression. J Trace Elem Med Biol. 2018;49:241–251. 10.1016/j.jtemb.2018.02.008 29452774

[55] Hashemipour M Kelishadi R Shapouri J. Effect of zinc supplementation on insulin resistance and components of the metabolic syndrome in prepubertal obese children. Hormones (Athens). 2009;8:279–285. 10.14310/horm.2002.1244 20045801

[56] Lobene AJ Kindler JM Jenkins NT. Zinc supplementation does not alter indicators of insulin secretion and sensitivity in black and white female adolescents. J Nutr. 2017;147:1296–1300. 10.3945/jn.117.248013 PMC5483963 28592518

[57] Gigot C Tung PW Wu H. Associations of childhood metals mixtures with fasting glucose and insulin resistance. Soc Sci Res Netw. 2025:5279726. doi:10.2139/ssrn.5279726.

[58] Desai G Queirolo EI Quattrin T. Association of blood arsenic concentrations with lipid markers in Uruguayan adolescents: exploring effect modification by body mass index and sex. Expo Health. 2026;18:33. 10.1007/s12403-026-00768-x PMC13079552 41994319

[59] Zuo J Huesker K Liu Y. Association of whole blood heavy metal concentrations with kidney function. Sci Rep. 2025;15:8370. 10.1038/s41598-025-93548-7 PMC11897145 40069484

[60] Castriota F Rieswijk L Dahlberg S. A state-of-the-science review of arsenic’s effects on glucose homeostasis in experimental models. Environ Health Perspect. 2020;128:16001. 10.1289/EHP4517 PMC7015542 31898917

[61] Abolli S Dehghani S Atlasi R. Arsenic and type 2 diabetes: revealing the environmental exposure relationship through effective factors—a systematic review. Res Eng. 2024;22:102054.

[62] Peng Q Harlow SD Park SK. Urinary arsenic and insulin resistance in US adolescents. Int J Hyg Environ Health. 2015;218:407–413. 10.1016/j.ijheh.2015.03.006 PMC4417072 25845984

[63] Paul SK Islam MS Hasibuzzaman MM. Higher risk of hyperglycemia with greater susceptibility in females in chronic arsenic-exposed individuals in Bangladesh. Sci Total Environ. 2019;668:1004–1012. 10.1016/j.scitotenv.2019.03.029 PMC6560360 31018442

[64] Lindberg AL Ekström EC Nermell B. Gender and age differences in the metabolism of inorganic arsenic in a highly exposed population in Bangladesh. Environ Res. 2008;106:110–120. 10.1016/j.envres.2007.08.011 17900557

[65] Martinez-Morata I Sobel M Tellez-Plaza M Navas-Acien A Howe CG Sanchez TR. A state-of-the-science review on metal biomarkers. Curr Environ Health Rep. 2023;10:215–249. 10.1007/s40572-023-00402-x PMC10822714 37337116

[66] ATSDR. Toxicological Profile for Arsenic. 2007. Available at: https://wwwn.cdc.gov/TSP/ToxProfiles/ToxProfiles.aspx?id=22&tid=3. Accessed June 14, 2024. 37184170

[67] Manley SE Luzio SD Stratton IM Wallace TM Clark PMS. Preanalytical, analytical, and computational factors affect homeostasis model assessment estimates. Diabetes Care. 2008;31:1877–1883. 10.2337/dc08-0097 PMC2518363 18535197

[68] Arslanian S Bacha F Grey M Marcus MD White NH Zeitler P. Evaluation and management of youth-onset type 2 diabetes: a position statement by the American diabetes association. Diabetes Care. 2018;41:2648–2668. 10.2337/dci18-0052 PMC7732108 30425094

