# Supplementary material for: Prospective associations of childhood metal mixtures with metabolic health in a Mexico City cohort
Source: Environ Epidemiol. 2026 Oct 26;10(6):e535. doi: 10.1097/EE9.0000000000000535 (PMC13713064; doi:10.1097/EE9.0000000000000535)
Supplement: Supplementary file 1 [file ee9-10-e535-s001.pdf]

Figure S1. Participants included in analyses.

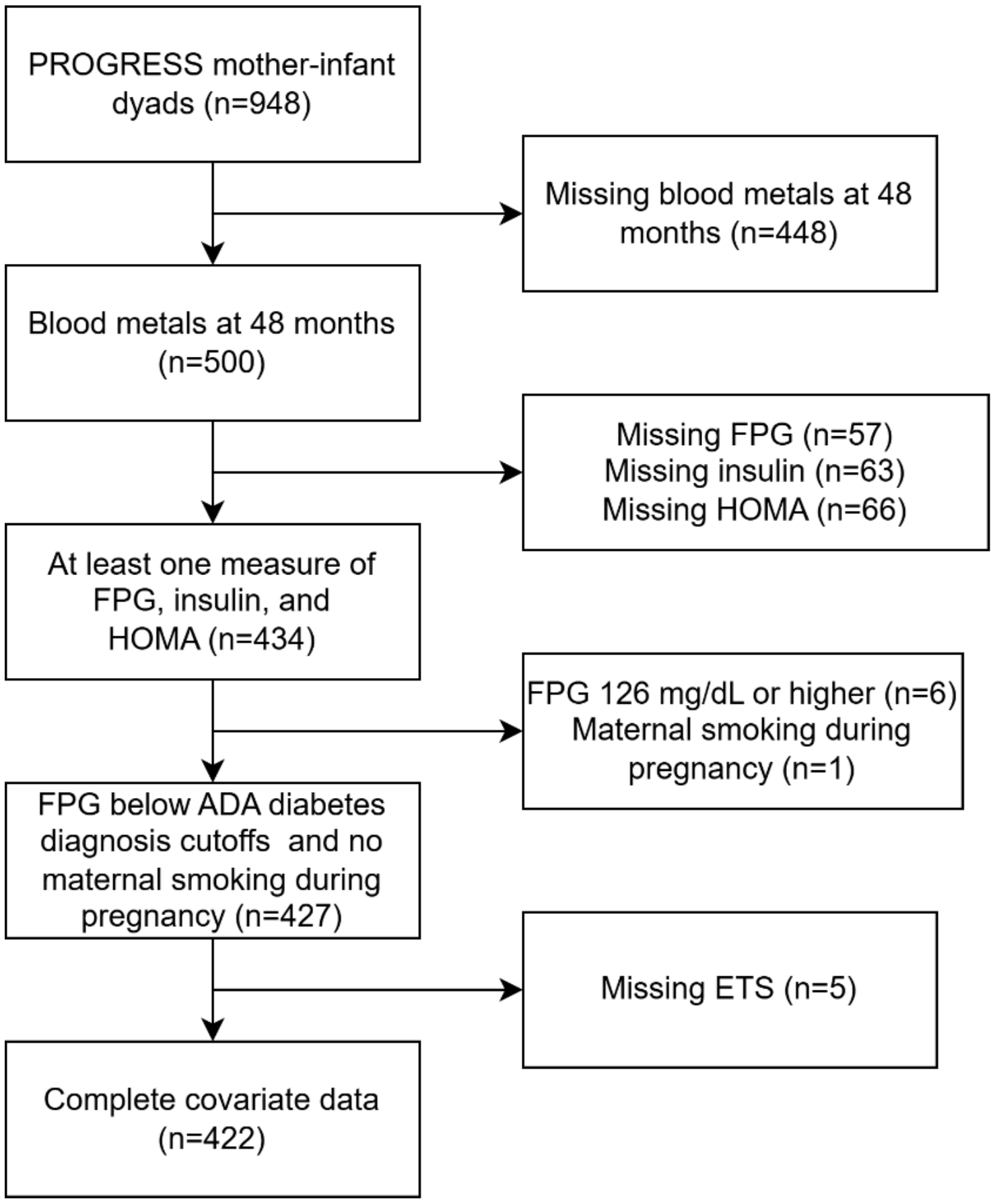

Figure S2. Directed acyclic graph of hypothesized relationships between exposures, covariates, and outcomes.

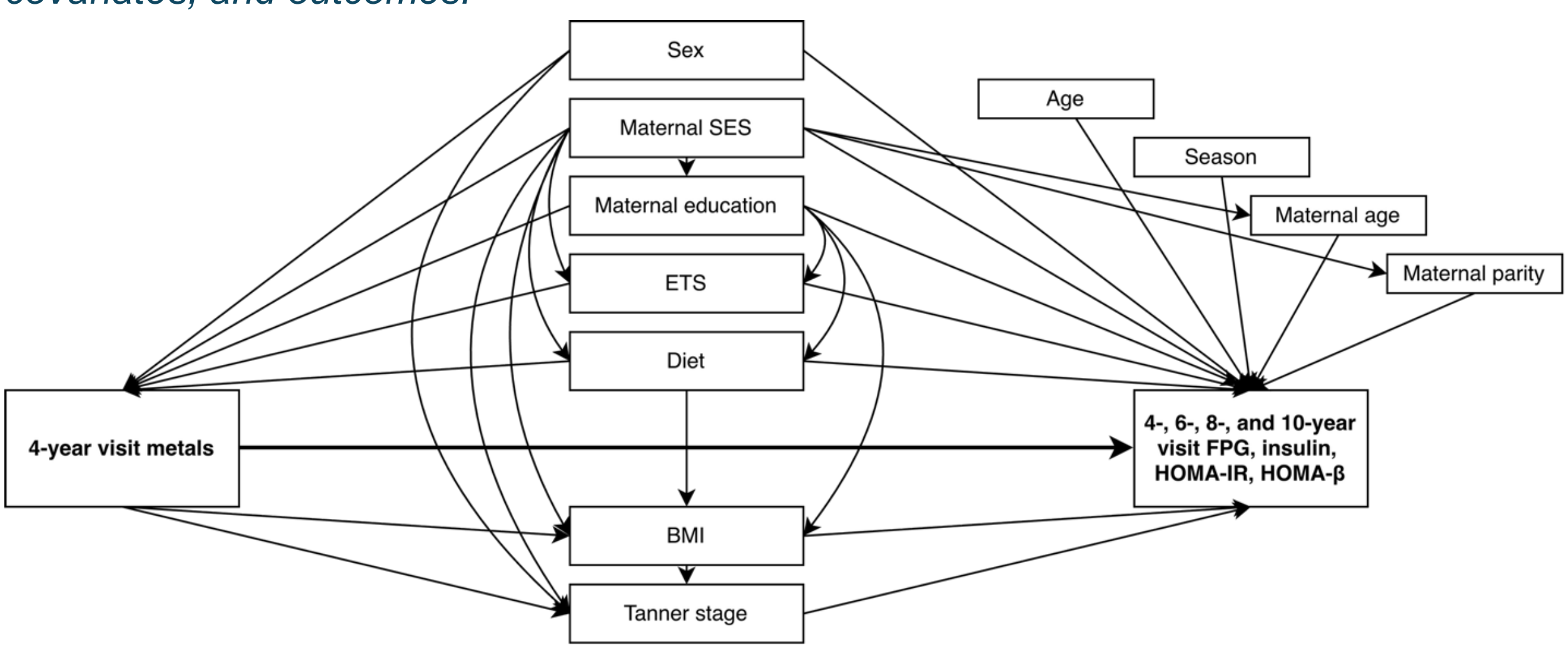

Note: We adjusted for diet (seafood, fast food, fruit and vegetable, and total energy intake) only in sensitivity analyses because of uncertainties around which dietary components contribute to metals exposure and metabolic health in the study population.

Figure S3. Correlations between blood metal concentrations (μg/L) at age 4-year visit (N=422).

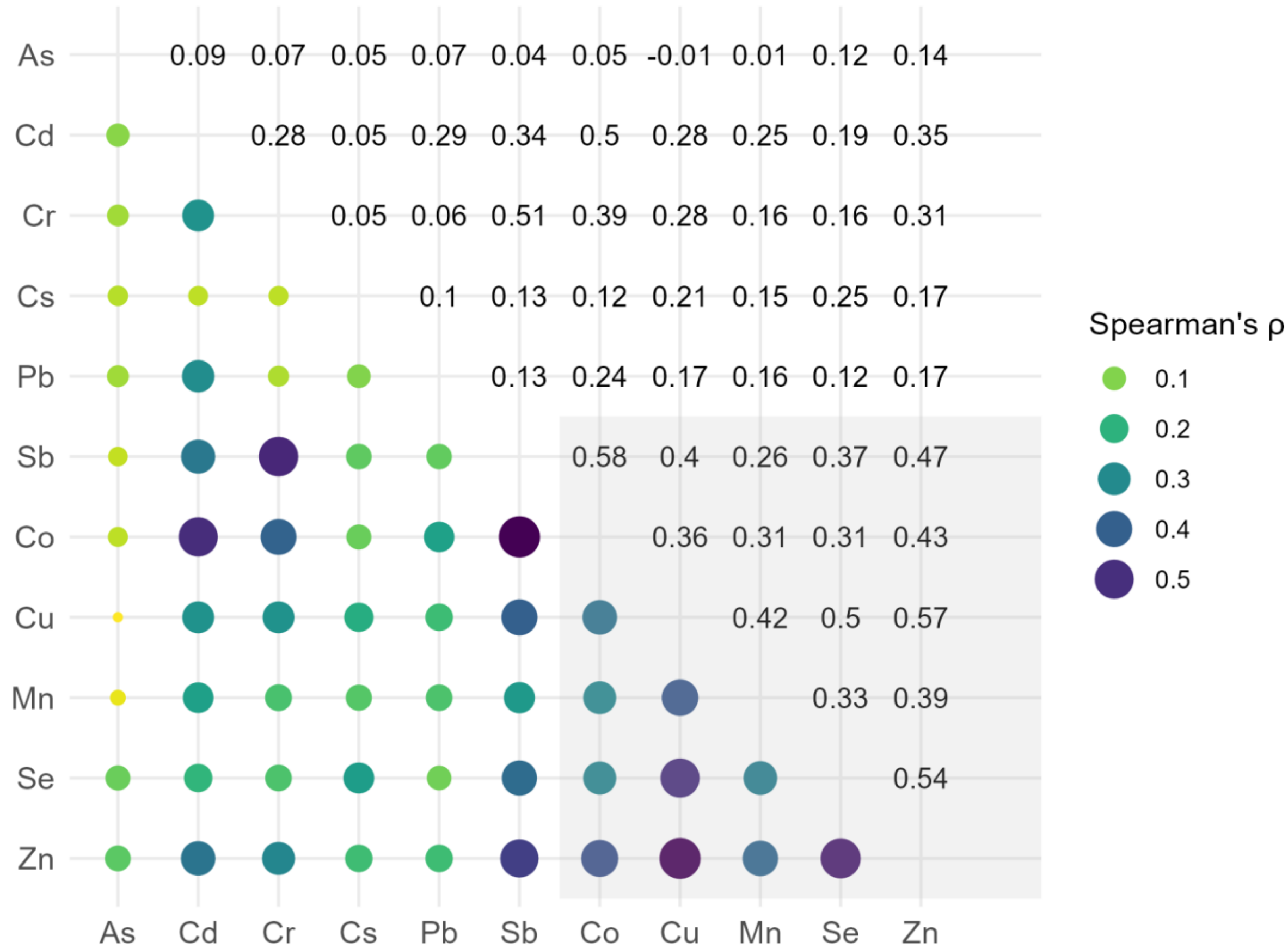

Figure S4. Correlations between metabolic outcomes.

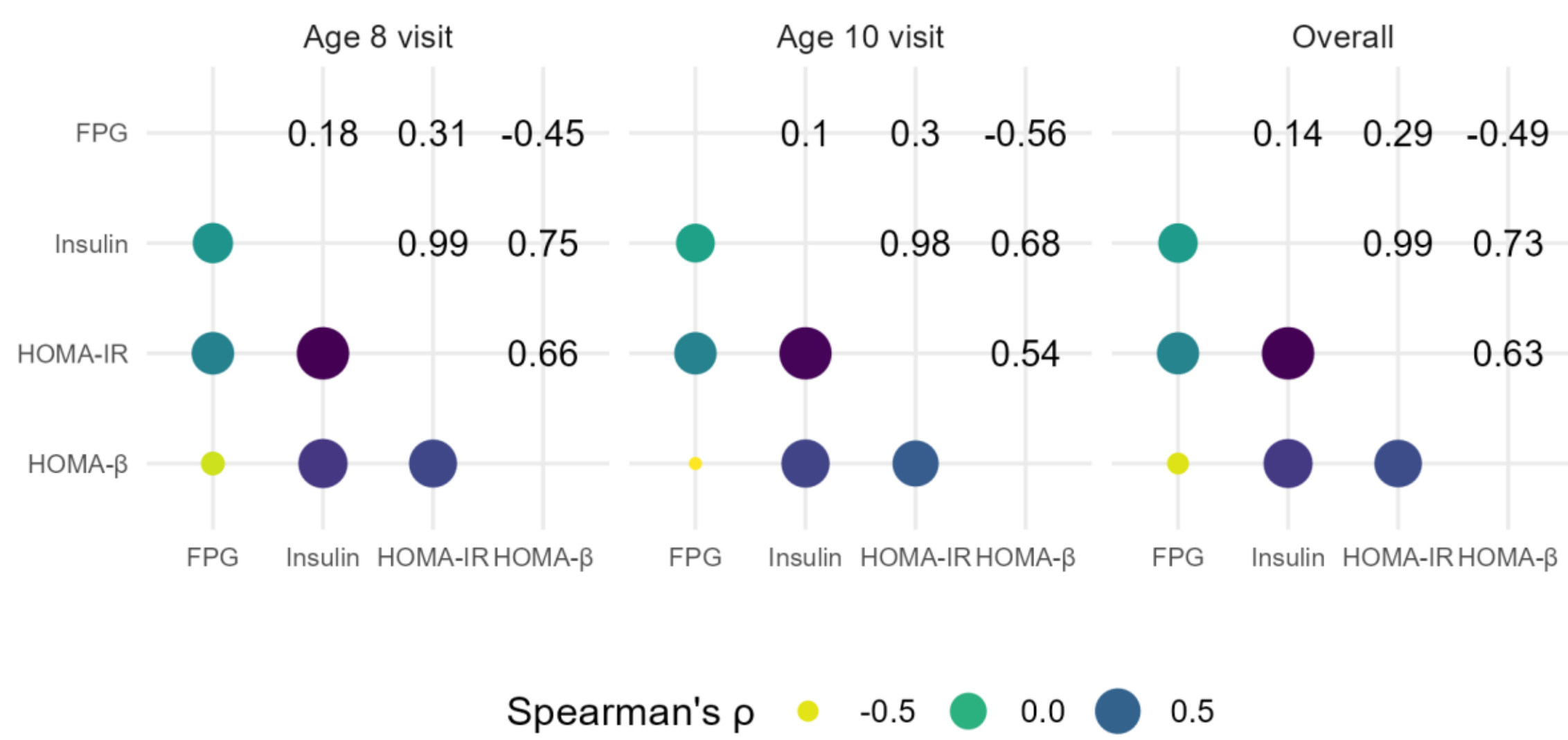

Figure S5. Associations of blood metal concentrations (age 4-year visit) with average metabolic outcomes at 4-, 6-, 8-, and 10-year visits, stratified by sex (N=422).

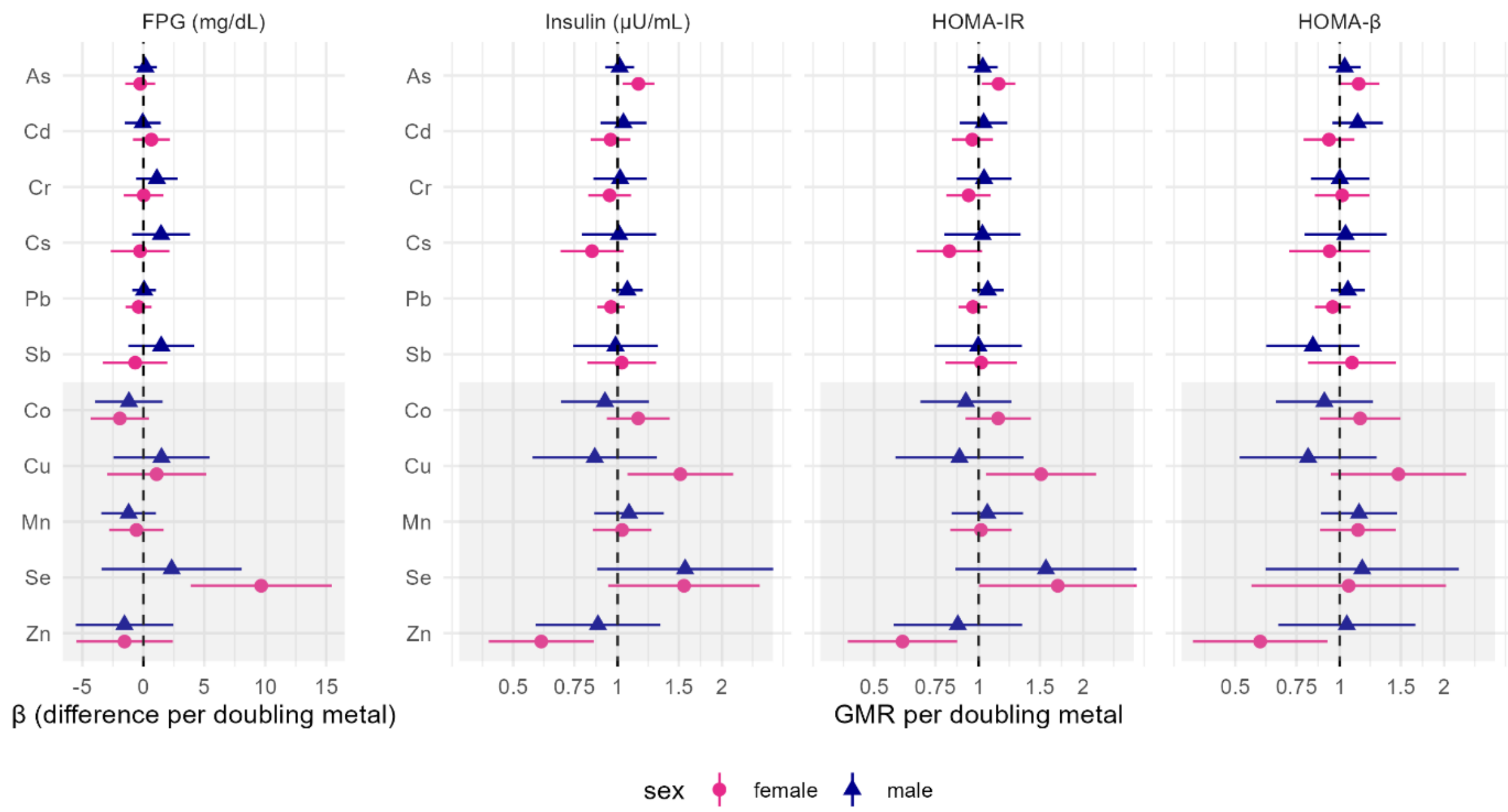

All models adjusted for maternal education, maternal socioeconomic status, exposure to environmental tobacco smoke defined by any smoker(s) in home at 4-year visit, age and season at outcome, and all other metals. P-values associated with interaction term for sex >0.05 for all metals and outcomes.

All models with interaction term for sex include the same 422 participants (1504 observations for FPG, 706 for insulin, 704 for HOMA-IR, and 698 for HOMA-β). For sex-stratified models, female-only models include 217 participants (770 observations for FPG, 362 insulin, 361 for HOMA-IR, 360 for HOMA-β); male-only models include 205 participants (734 observations for FPG, 344 for insulin, 343 for HOMA-IR, 338 for HOMA-β).

Figure S6. Associations of blood metal concentrations (age 4-year visit) with metabolic outcomes at 4-, 6-, 8-, and 10-year visits.

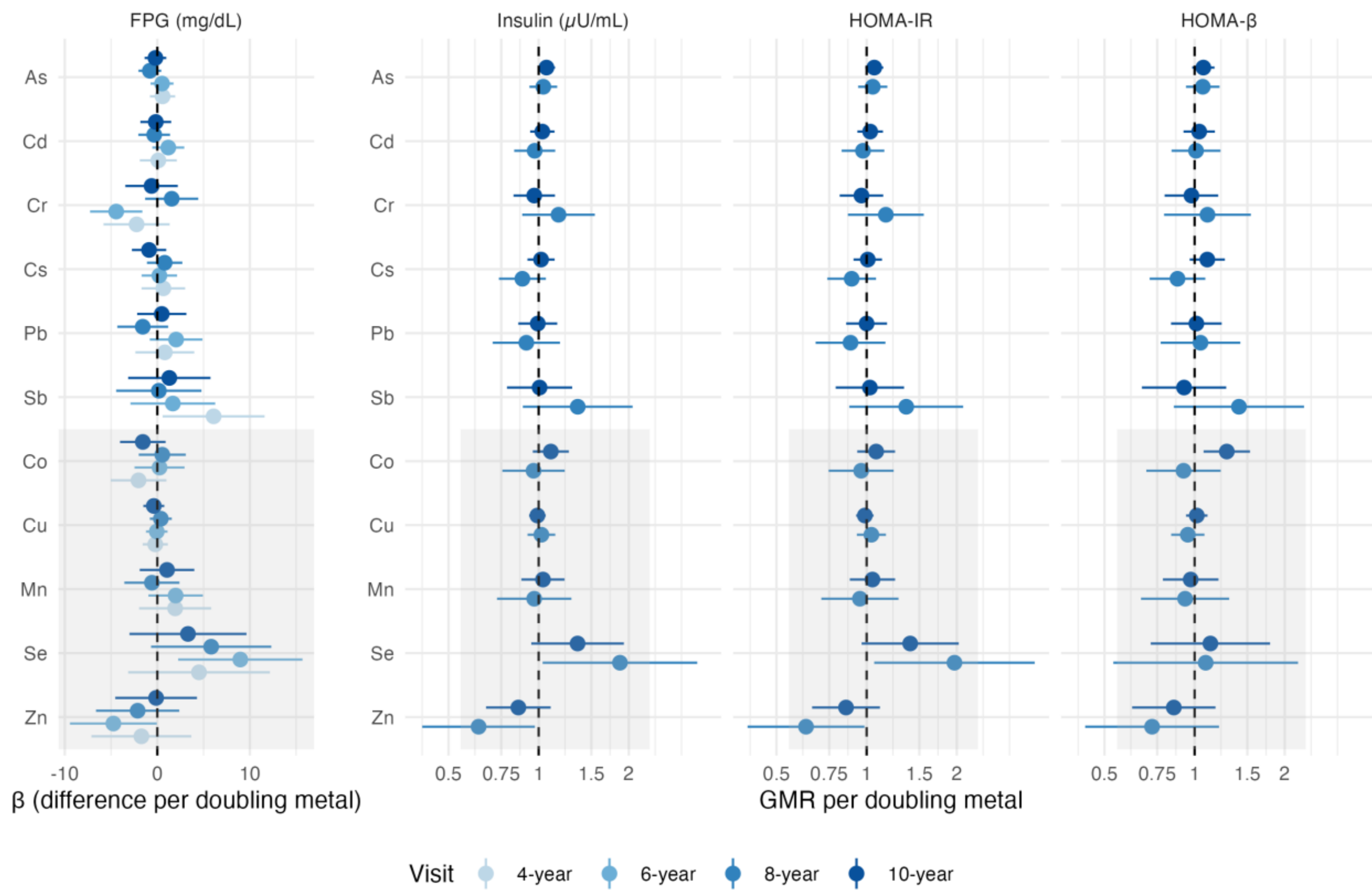

All models adjusted for sex, maternal education, maternal socioeconomic status, exposure to environmental tobacco smoke defined by any smoker(s) in home at 4-year visit, age and season at outcome, and all other metals. Circles represent point estimates; lines 95% confidence intervals. Insulin, HOMA-IR, and HOMA-β were log_2-tranformed, so associations were exponentiated to geometric mean ratios (GMRs).

Separate linear regression models were fit for each outcome–visit combination. FPG was available at the 4-, 6-, 8-, and 10-year visits (n=377, 328, 399, and 400 participants, respectively); insulin was assayed only at the 8- and 10-year visits, so insulin, HOMA-IR, and HOMA-β models were restricted to those visits (insulin: n=306 and 400; HOMA-IR: n=304 and 400; HOMA-β: n=302 and 396).

Table S1. Participant characteristics: original cohort vs. study sample.

Characteristic	PROGRESS cohort N=948[a]	Study population N=422[a]	p-value[b]
Baseline			
Sex			0.5
Female	498 (53%)	217 (51%)	
Male	450 (47%)	205 (49%)	
SES			0.2
Low	486 (51%)	229 (54%)	
Medium	356 (38%)	153 (36%)	
High	106 (11%)	40 (9.5%)	
Maternal education			0.2
<high school	385 (41%)	173 (41%)	
High school	334 (35%)	158 (37%)	
>high school	229 (24%)	91 (22%)	
Mother's age (2nd trimester)	27.0 (23.0, 31.0)	27.0 (23.0, 31.0)	0.7
Mother's parity			0.9
Primiparous	355 (37%)	159 (38%)	
Multiparous	593 (63%)	263 (62%)	
ETS	88 (15%)	56 (13%)	0.14
Unknown	348	0	
4-year visit			
Age	4.77 (4.36, 5.11)	4.75 (4.35, 5.10)	0.10
Unknown	340	0	
Season			0.3
Cold/dry	175 (29%)	120 (28%)	
Warm/dry	114 (19%)	86 (20%)	
Rainy	319 (52%)	216 (51%)	
Unknown	340	0	
zBMI	0.14 (-0.47, 0.72)	0.12 (-0.50, 0.70)	0.3
Unknown	340	0	
FFQ: Total energy intake (kcal/day)	1,548 (1,261, 1,961)	1,529 (1,278, 1,961)	0.9
Unknown	355	4	
FFQ: Seafood (g/day)	5 (0, 13)	5 (0, 12)	0.8
Unknown	355	4	
FFQ: Fruit and vegetables (g/day)	213 (118, 378)	219 (118, 391)	0.7
Unknown	355	4	
FFQ: fast food (g/day)	0 (0, 13)	0 (0, 7)	0.12
Unknown	355	4	

[a]N (%), median (IQR). [b]P-value associated with Pearson's Chi-squared test or Wilcoxon rank sum test, as appropriate, for difference between PROGRESS cohort included versus excluded (among those with known values for that characteristic). SES = socioeconomic status; ETS = exposure to environmental tobacco smoke defined by any smoker(s) in home at 4-year visit; zBMI = BMI-for-age (z-score); FFQ = food frequency questionnaire.

Table S2. Intraclass correlation coefficients for metabolic outcomes, estimate (95% confidence intervals).

	4-, 6-, 8- and 10-year visits	8- and 10-year visits only
FPG (mg/dL)	0.14 (0.088, 0.19)	0.15 (0.047, 0.25)
Serum insulin (μU/mL)		0.27 (0.15, 0.37)
HOMA-IR		0.24 (0.13, 0.35)
HOMA-β		0.23 (0.11, 0.34)

Table S3. Associations of individual blood metal concentrations with average metabolic outcomes from generalized additive mixed models (N=422).

	FPG				Insulin				HOMA-IR				HOMA-β			
Metal	EDF	F-statistic	p-value	p-value (adj.)	EDF	F-statistic	p-value	p-value (adj.)	EDF	F-statistic	p-value	p-value (adj.)	EDF	F-statistic	p-value	p-value (adj.)
As	1.9	0.8	0.45	>0.99	1.1	2.2	0.12	>0.99	1.4	1.5	0.15	>0.99	1	2.6	0.11	>0.99
Cd	1	0.0094	0.92	>0.99	1	0.017	0.9	>0.99	1	0.014	0.91	>0.99	1	0.12	0.73	>0.99
Co	1	1.1	0.3	>0.99	1	0.26	0.61	>0.99	1	0.18	0.67	>0.99	1	0.0087	0.93	>0.99
Cr	1	0.58	0.45	>0.99	1	0.13	0.71	>0.99	1	0.14	0.71	>0.99	1.4	0.14	0.85	>0.99
Cs	1	1.9	0.17	>0.99	1	0.0011	0.97	>0.99	1	0.0062	0.94	>0.99	1	0.079	0.78	>0.99
Cu	2	2.6	0.081	0.89	1	1.3	0.26	>0.99	1	1.4	0.24	>0.99	1	0.39	0.53	>0.99
Mn	1	0.012	0.91	>0.99	1	1.3	0.26	>0.99	1	0.94	0.33	>0.99	1.9	1.3	0.18	>0.99
Pb	1	0.088	0.77	>0.99	1	0.34	0.56	>0.99	1.1	0.32	0.56	>0.99	1	0.031	0.86	>0.99
Sb	1	0.1	0.75	>0.99	1	0.0032	0.96	>0.99	1	0.004	0.95	>0.99	1	0.19	0.66	>0.99
Se	1	8.7	**0.0033**	**0.036**	1	5.2	**0.022**	0.25	1	5.8	**0.016**	0.18	1	0.55	0.46	>0.99
Zn	1	0.042	0.84	>0.99	1	0.083	0.77	>0.99	1	0.06	0.81	>0.99	1	0.18	0.67	>0.99

All models adjusted for sex, maternal education, maternal socioeconomic status, exposure to environmental tobacco smoke defined by any smoker(s) in home at 4-year visit, and age and season at outcome. EDF = effective degrees of freedom (EDF= 1 is a linear relationship and higher values indicate increasingly complex, nonlinear curves. F-statistic tests whether the smooth term explains a significant amount of model variance, and p-value is the probability of observing an F-statistic as large or larger if the smooth term has no true effect; p-value (adj.) is the p-value adjusted for multiple comparisons across 11 metals, using the Bonferroni method. All models include the same 422 participants (1504 observations for FPG, 706 for insulin, 704 for HOMA-IR, and 698 for HOMA-β).

Table S4. Associations of average metabolic outcomes at 4-, 6-, 8-, and 10-year visits with doubling individual blood metals at age 4 visit (N=422).

	Metal	Association (95% CI)	p-value	p-value (adj.)
Fasting glucose	As	-0.083 (-0.81, 0.65)	0.82	>0.99
	Cd	0.24 (-0.81, 1.3)	0.66	>0.99
	Co	-1.7 (-3.4, 0.1)	0.065	0.72
	Cr	0.39 (-0.77, 1.5)	0.51	>0.99
	Cs	0.61 (-1.1, 2.3)	0.47	>0.99
	Cu	1.2 (-1.6, 4)	0.39	>0.99
	Mn	-0.66 (-2.2, 0.89)	0.4	>0.99
	Pb	-0.077 (-0.79, 0.63)	0.83	>0.99
	Sb	0.87 (-0.97, 2.7)	0.35	>0.99
	Se	**5.8 (1.8, 9.8)**	**0.0041**	**0.045**
	Zn	-2.1 (-4.9, 0.6)	0.13	>0.99
Insulin	As	1.1 (0.99, 1.1)	0.11	>0.99
	Cd	0.99 (0.9, 1.1)	0.86	>0.99
	Co	1 (0.88, 1.2)	0.62	>0.99
	Cr	0.97 (0.87, 1.1)	0.54	>0.99
	Cs	0.95 (0.81, 1.1)	0.49	>0.99
	Cu	1.1 (0.83, 1.4)	0.57	>0.99
	Mn	1.1 (0.91, 1.2)	0.48	>0.99
	Pb	1 (0.95, 1.1)	0.7	>0.99
	Sb	1 (0.85, 1.2)	0.84	>0.99
	Se	**1.6 (1.1, 2.3)**	**0.018**	0.2
	Zn	**0.75 (0.58, 0.98)**	**0.034**	0.37
HOMA-IR	As	1.1 (0.99, 1.1)	0.11	>0.99
	Cd	0.99 (0.89, 1.1)	0.87	>0.99
	Co	1 (0.87, 1.2)	0.67	>0.99
	Cr	0.96 (0.86, 1.1)	0.53	>0.99
	Cs	0.94 (0.79, 1.1)	0.44	>0.99
	Cu	1.1 (0.83, 1.4)	0.53	>0.99
	Mn	1 (0.89, 1.2)	0.62	>0.99
	Pb	1 (0.95, 1.1)	0.66	>0.99
	Sb	1 (0.85, 1.2)	0.82	>0.99
	Se	**1.6 (1.1, 2.4)**	**0.013**	0.14
	Zn	**0.74 (0.57, 0.98)**	**0.035**	0.38
HOMA-β	As	1.1 (0.99, 1.2)	0.094	>0.99
	Cd	1 (0.9, 1.1)	0.84	>0.99
	Co	1 (0.84, 1.2)	0.83	>0.99
	Cr	1 (0.88, 1.1)	0.98	>0.99
	Cs	1 (0.83, 1.2)	0.94	>0.99
	Cu	1.1 (0.78, 1.5)	0.68	>0.99
	Mn	1.1 (0.95, 1.3)	0.17	>0.99
	Pb	1 (0.92, 1.1)	0.96	>0.99
	Sb	0.96 (0.78, 1.2)	0.7	>0.99
	Se	1.1 (0.73, 1.8)	0.57	>0.99
	Zn	0.81 (0.59, 1.1)	0.19	>0.99

For FPG, association represents the adjusted average change (in mg/dL) per quartile increase in metals; for insulin, HOMA-IR, and HOMA-β, the joint association represents the adjusted GMR per quartile increase in metals. p-value (adj.) is the p-value adjusted for multiple comparisons across 11 metals, using the Bonferroni method. All models adjusted for sex, maternal education, maternal socioeconomic status, exposure to environmental tobacco smoke defined by any smoker(s) in home at 4-year visit, age and season at outcome, and all other metals. All models include the same 422 participants (1504 observations for FPG, 706 for insulin, 704 for HOMA-IR, and 698 for HOMA-β).

Table S5. Difference in average FPG (md/dL) at 4-, 6-, 8-, and 10-year visits and 95% confidence interval associated with doubling individual blood metal concentrations at age 4-year visit.

	Model 1	Model 1, excluding age 4-year visit	Model 2 (+BMI)	Model 3 (+puberty)	Model 4 (+diet)	Model 5 (+maternal age and parity)
As	-0.083 (-0.81, 0.65)	-0.24 (-1.0, 0.55)	-0.12 (-0.85, 0.6)	-0.47 (-1.4, 0.45)	-0.066 (-0.81, 0.68)	-0.09 (-0.82, 0.64)
Cd	0.24 (-0.8, 1.3)	0.26 (-0.85, 1.4)	0.33 (-0.7, 1.4)	-0.43 (-1.7, 0.89)	0.27 (-0.79, 1.3)	0.28 (-0.79, 1.3)
Cr	0.39 (-0.77, 1.5)	-1.2 (-3.0, 0.65)	0.34 (-0.8, 1.5)	-0.51 (-1.9, 0.93)	0.42 (-0.75, 1.6)	0.4 (-0.76, 1.6)
Cs	0.61 (-1.1, 2.3)	-0.14 (-1.4, 1.1)	0.5 (-1.2, 2.2)	-0.46 (-2.6, 1.6)	0.47 (-1.2, 2.2)	0.61 (-1.1, 2.3)
Pb	-0.077 (-0.78, 0.63)	0.25 (-1.5, 2.0)	-0.038 (-0.74, 0.67)	-0.054 (-0.95, 0.84)	-0.11 (-0.84, 0.62)	-0.065 (-0.78, 0.65)
Sb	0.87 (-0.96, 2.7)	0.19 (-2.7, 3.1)	0.79 (-1, 2.6)	0.42 (-1.9, 2.7)	0.93 (-0.93, 2.8)	0.84 (-1, 2.7)
Co	-1.7 (-3.4, 0.096)	-0.25 (-1.9, 1.4)	**-1.7 (-3.5, -0.0076)**	0.15 (-2, 2.3)	-1.6 (-3.4, 0.16)	-1.7 (-3.5, 0.06)
Cu	1.2 (-1.6, 4)	0.0084 (-0.75, 0.76)	0.92 (-1.8, 3.7)	0.26 (-3.2, 3.7)	1.6 (-1.3, 4.4)	1.2 (-1.6, 4)
Mn	-0.66 (-2.2, 0.89)	0.66 (-1.3, 2.6)	-0.98 (-2.5, 0.57)	0.17 (-1.8, 2.1)	-0.56 (-2.1, 1)	-0.69 (-2.2, 0.87)
Se	**5.8 (1.9, 9.7)**	**6.1 (1.9, 10)**	**5.4 (1.5, 9.3)**	4.5 (-0.48, 9.4)	**5.9 (2, 9.9)**	**5.8 (1.9, 9.8)**
Zn	-2.1 (-4.9, 0.6)	-2.0 (-4.9, 0.94)	-1.4 (-4.1, 1.4)	-1.2 (-4.6, 2.2)	-2.3 (-5.1, 0.46)	-2.2 (-4.9, 0.59)

Model 1: adjusted for sex, maternal education, maternal socioeconomic status, exposure to environmental tobacco smoke defined by any smoker(s) in home at 4-year visit, age and season at outcome, and all other metals (n=422 participants, 1504 observations).
Model 1, excluding age 4-year visit (n=422 participants, 1127 observations).
Model 2: adjusted for covariates in Model 1 plus zBMI at outcome (n=422 participants, 1502 observations).
Model 3: adjusted for covariates in Model 1 plus puberty status at outcome (n=420 participants, 779 observations).
Model 4: adjusted for covariates in Model 1 plus for diet (seafood, fast food, fruit and vegetable, and total energy intake) at 4-year visit (n=418 participants, 1490 observations).
Model 5: adjusted for covariates in Model 1 plus maternal characteristics (age and parity) (n=422 participants, 1504 observations).

Table S6. Geometric mean ratio and 95% confidence interval of average metabolic outcomes at 4-, 6-, 8-, and 10-year visits associated with doubling individual blood metals at age 4 visit.

		Model 1	Model 2 (+BMI)	Model 3 (+puberty)	Model 4 (+diet)	Model 5 (+maternal age and parity)
Insulin	As	1.1 (0.99, 1.1)	1 (0.96, 1.1)	1.1 (0.98, 1.1)	1.1 (0.98, 1.1)	1.1 (0.99, 1.1)
	Cd	0.99 (0.9, 1.1)	1 (0.93, 1.1)	1 (0.9, 1.1)	1 (0.9, 1.1)	1 (0.91, 1.1)
	Cr	0.97 (0.87, 1.1)	0.97 (0.89, 1.1)	0.96 (0.86, 1.1)	0.96 (0.86, 1.1)	0.97 (0.87, 1.1)
	Cs	0.95 (0.81, 1.1)	0.92 (0.81, 1)	0.94 (0.8, 1.1)	0.93 (0.79, 1.1)	0.94 (0.8, 1.1)
	Pb	1 (0.95, 1.1)	1 (0.98, 1.1)	1 (0.94, 1.1)	1 (0.94, 1.1)	1 (0.95, 1.1)
	Sb	1 (0.85, 1.2)	0.98 (0.86, 1.1)	1 (0.85, 1.2)	1 (0.85, 1.2)	1 (0.85, 1.2)
	Co	1 (0.88, 1.2)	0.98 (0.86, 1.1)	1 (0.87, 1.2)	1.1 (0.89, 1.2)	1 (0.88, 1.2)
	Cu	1.1 (0.83, 1.4)	1 (0.85, 1.3)	1 (0.8, 1.3)	1.1 (0.81, 1.4)	1.1 (0.82, 1.4)
	Mn	1.1 (0.91, 1.2)	0.93 (0.83, 1)	1.1 (0.92, 1.2)	1.1 (0.92, 1.2)	1.1 (0.91, 1.2)
	Se	**1.6 (1.1, 2.3)**	1.3 (0.95, 1.7)	**1.5 (1.1, 2.2)**	**1.6 (1.1, 2.3)**	**1.6 (1.1, 2.3)**
	Zn	**0.75 (0.58, 0.98)**	1 (0.83, 1.3)	0.78 (0.6, 1)	**0.75 (0.58, 0.98)**	**0.74 (0.57, 0.97)**
HOMA-IR	As	1.1 (0.99, 1.1)	1 (0.96, 1.1)	1.1 (0.98, 1.1)	1.1 (0.98, 1.1)	1.1 (0.99, 1.1)
	Cd	0.99 (0.89, 1.1)	1 (0.92, 1.1)	1 (0.9, 1.1)	1 (0.9, 1.1)	1 (0.9, 1.1)
	Cr	0.96 (0.86, 1.1)	0.97 (0.88, 1.1)	0.96 (0.86, 1.1)	0.96 (0.85, 1.1)	0.97 (0.86, 1.1)
	Cs	0.94 (0.79, 1.1)	0.91 (0.8, 1)	0.93 (0.79, 1.1)	0.92 (0.78, 1.1)	0.93 (0.79, 1.1)
	Pb	1 (0.95, 1.1)	1 (0.98, 1.1)	1 (0.94, 1.1)	1 (0.94, 1.1)	1 (0.95, 1.1)
	Sb	1 (0.85, 1.2)	0.99 (0.85, 1.1)	1 (0.85, 1.2)	1 (0.85, 1.2)	1 (0.84, 1.2)
	Co	1 (0.87, 1.2)	0.98 (0.86, 1.1)	1 (0.86, 1.2)	1 (0.88, 1.2)	1 (0.87, 1.2)
	Cu	1.1 (0.83, 1.4)	1.1 (0.85, 1.3)	1.1 (0.8, 1.4)	1.1 (0.82, 1.4)	1.1 (0.83, 1.4)
	Mn	1 (0.89, 1.2)	0.92 (0.81, 1)	1.1 (0.9, 1.2)	1.1 (0.9, 1.2)	1 (0.89, 1.2)
	Se	**1.6 (1.1, 2.4)**	1.3 (0.98, 1.8)	**1.6 (1.1, 2.4)**	**1.7 (1.1, 2.5)**	**1.7 (1.1, 2.4)**
	Zn	**0.74 (0.57, 0.98)**	1 (0.81, 1.3)	0.77 (0.59, 1)	**0.74 (0.56, 0.98)**	**0.74 (0.56, 0.97)**
HOMA-β	As	1.1 (0.99, 1.2)	1 (0.96, 1.1)	1.1 (0.99, 1.2)	1.1 (0.98, 1.2)	1.1 (0.99, 1.2)
	Cd	1 (0.9, 1.1)	1 (0.92, 1.1)	1 (0.91, 1.1)	1 (0.9, 1.1)	1 (0.91, 1.2)
	Cr	1 (0.88, 1.1)	1 (0.9, 1.1)	1 (0.89, 1.1)	0.99 (0.87, 1.1)	1 (0.88, 1.1)
	Cs	1 (0.83, 1.2)	0.98 (0.83, 1.2)	1 (0.83, 1.2)	1 (0.83, 1.2)	1 (0.83, 1.2)
	Pb	1 (0.92, 1.1)	1 (0.94, 1.1)	0.99 (0.92, 1.1)	0.99 (0.92, 1.1)	1 (0.92, 1.1)
	Sb	0.96 (0.78, 1.2)	0.93 (0.78, 1.1)	0.94 (0.76, 1.2)	0.95 (0.77, 1.2)	0.95 (0.77, 1.2)
	Co	1 (0.84, 1.2)	0.97 (0.81, 1.2)	1 (0.83, 1.2)	1 (0.84, 1.3)	1 (0.83, 1.2)
	Cu	1.1 (0.78, 1.5)	1 (0.78, 1.4)	1 (0.75, 1.4)	1 (0.75, 1.4)	1.1 (0.78, 1.4)
	Mn	1.1 (0.95, 1.3)	1 (0.86, 1.2)	1.1 (0.93, 1.3)	1.1 (0.95, 1.3)	1.1 (0.95, 1.3)
	Se	1.1 (0.73, 1.8)	0.95 (0.64, 1.4)	1.1 (0.73, 1.7)	1.1 (0.72, 1.8)	1.1 (0.74, 1.8)
	Zn	0.81 (0.59, 1.1)	1.1 (0.81, 1.4)	0.86 (0.63, 1.2)	0.82 (0.6, 1.1)	0.81 (0.59, 1.1)

Model 1: adjusted for sex, maternal education, maternal socioeconomic status, exposure to environmental tobacco smoke defined by any smoker(s) in home at 4-year visit, age and season at outcome, and all other metals (n=422 participants for all outcomes; 706, 704, and 698 observations for insulin, HOMA-IR, and HOMA-β, respectively).
Model 2: adjusted for covariates in Model 1 plus zBMI at outcome (n=422 participants for all outcomes; 705, 703, and 697 observations for insulin, HOMA-IR, and HOMA-β, respectively).
Model 3: adjusted for covariates in Model 1 plus puberty status at outcome (n=420 participants for all outcomes; 689, 687, and 681 observations for insulin, HOMA-IR, and HOMA-β, respectively).
Model 4: adjusted for covariates in Model 1 plus for diet (seafood, fast food, fruit and vegetable, and total energy intake) at 4-year visit (n=418 participants for all outcomes; 699, 697, and 691 observations for insulin, HOMA-IR, and HOMA-β, respectively).
Model 5: adjusted for covariates in Model 1 plus maternal characteristics (age and parity) (n=422 participants for all outcomes; 706, 704, and 698 observations for insulin, HOMA-IR, and HOMA-β, respectively).

Table S7. Quantile g-computation estimates (difference in average FPG at 4-, 6-, 8-, and 10-year visits and 95% confidence intervals; weights) for simultaneous quartile increase in all log_2-transformed age 4-year visit blood metal concentrations, stratified by sex.

Female				Male				p-int.
Adjusted change per quartile increase	Metal	Negative weight	Positive weight	Adjusted change per quartile increase	Metal	Negative weight	Positive weight	
0.38 (-1.1, 1.8)				**1.7 (0.29, 3.2)**				0.18
	Se	-	0.76		As	-	0.31	
	Sb	-	0.15		Mn	-	0.16	
	Cd	-	0.048		Cs	-	0.16	
	Pb	-	0.039		Cr	-	0.15	
	Co	0.64	-		Cu	-	0.073	
	Zn	0.14	-		Sb	-	0.066	
	Cr	0.095	-		Se	-	0.054	
	Cu	0.053	-		Pb	-	0.02	
	Mn	0.036	-		Cd	0.61	-	
	As	0.022	-		Zn	0.31	-	
	Cs	0.014	-		Co	0.081	-	

All models adjusted for sex, maternal education, maternal socioeconomic status, exposure to environmental tobacco smoke defined by any smoker(s) in home at 4-year visit, and age and season at outcome. Weights represent the proportion of the positive or negative partial effect for each component (metal) in the mixture on metabolic outcomes; negative weights and positive weights each sum to 1.

Model with interaction term for sex included 422 participants (1504 observations). Female-only model included 217 participants (770 observations); male-only model included 205 participants (734 observations).

Table S8. Quantile g-computation estimates (joint associations and 95% confidence intervals; weights) for simultaneous quartile increase in all log2-transformed age 4-year visit blood metal concentrations, stratified by sex.

Outcome (age 4-10 visits)	Female Adjusted GMR per quartile increase	Metal	Negative weight	Positive weight	Male Adjusted GMR per quartile increase	Metal	Negative weight	Positive weight	p-int
Insulin	1.09 (0.953, 1.24)				1.08 (0.922, 1.26)				0.85
		Cu	-	0.27		Se	-	0.5	
		As	-	0.21		As	-	0.19	
		Se	-	0.19		Pb	-	0.1	
		Co	-	0.17		Cs	-	0.078	
		Mn	-	0.074		Mn	-	0.077	
		Sb	-	0.052		Cd	-	0.056	
		Cr	-	0.029		Zn	0.33	-	
		Pb	-	0.005		Sb	0.28	-	
		Zn	0.48	-		Co	0.16	-	
		Cs	0.32	-		Cu	0.14	-	
		Cd	0.21	-		Cr	0.089	-	
HOMA-IR	1.09 (0.951, 1.24)				1.1 (0.953, 1.27)				0.93
		Cu	-	0.25		Se	-	0.41	
		Se	-	0.21		As	-	0.29	
		As	-	0.2		Pb	-	0.13	
		Co	-	0.14		Mn	-	0.092	
		Sb	-	0.074		Cs	-	0.058	
		Mn	-	0.065		Cd	-	0.026	
		Pb	-	0.052		Zn	0.32	-	
		Cr	-	0.016		Sb	0.3	-	
		Zn	0.47	-		Co	0.27	-	
		Cs	0.33	-		Cu	0.065	-	
		Cd	0.21	-		Cr	0.045	-	
HOMA-β	1.11 (0.929, 1.32)				1.01 (0.843, 1.2)				0.55
		Cu	-	0.34		Cd	-	0.44	
		Co	-	0.25		Se	-	0.33	
		As	-	0.2		Cs	-	0.084	
		Mn	-	0.07		Cr	-	0.076	
		Cr	-	0.069		Pb	-	0.06	
		Se	-	0.033		Zn	-	0.014	
		Sb	-	0.032		Sb	0.53	-	
		Zn	0.6	-		Co	0.24	-	
		Pb	0.21	-		Cu	0.12	-	
		Cd	0.13	-		As	0.088	-	
		Cs	0.063	-		Mn	0.025	-	

All models adjusted for sex, maternal education, maternal socioeconomic status, exposure to environmental tobacco smoke defined by any smoker(s) in home at 4-year visit, and age and season at outcome. Weights represent the proportion of the positive or negative partial effect for each component (metal) in the mixture on metabolic outcomes; negative weights and positive weights each sum to 1.

All models with interaction term for sex include the same 422 participants (706 observations for insulin, 704 for HOMA-IR, and 698 for HOMA-β). For sex-stratified models, female-only models include 217 participants (362 observations for insulin, 361 for HOMA-IR, 360 for HOMA-β); male-only models include 205 participants (344 observations for insulin, 343 for HOMA-IR, 338 for HOMA-β).

Table S9. Quantile g-computation estimates (difference in average FPG at 6-, 8-, and 10-year visits and 95% confidence intervals; weights) for simultaneous quartile increase in all log_2-transformed age 4-year visit blood metal concentrations (n=422 participants, 1127 observations).

Adjusted change per quartile increase	Metal	Negative weight	Positive weight
0.75 (-0.34, 1.8)			
	Se	-	0.6
	As	-	0.14
	Mn	-	0.12
	Pb	-	0.12
	Cs	-	0.026
	Zn	0.39	-
	Co	0.29	-
	Cd	0.13	-
	Cr	0.1	-
	Sb	0.062	-
	Cu	0.026	-

All models adjusted for sex, maternal education, maternal socioeconomic status, exposure to environmental tobacco smoke defined by any smoker(s) in home at 4-year visit, and age and season at outcome. Weights represent the proportion of the positive or negative partial effect for each component (metal) in the mixture on metabolic outcomes; negative weights and positive weights each sum to 1.

Table S10. Additionally adjusted quantile g-computation estimates (joint associations and 95% confidence intervals) for simultaneous quartile increase in all log_2-transformed age 4-year visit blood metal concentrations.

Outcome (age 4-10 years)	Model 1	Model 2 (+BMI)	Model 3 (+puberty)	Model 4 (+diet)	Model 5 (+maternal age and parity)
FPG	0.93 (-0.084, 1.9)	0.93 (-0.072, 1.94)	0.57 (-0.86, 1.99)	0.90 (-0.11, 1.9)	0.93 (-0.041, 1.91)
Insulin	**1.10 (1.01, 1.22)**	**1.11 (1.01, 1.21)**	1.10 (1.00, 1.21)	1.10 (1.00, 1.21)	1.10 (1.00, 1.22)
HOMA-IR	**1.11 (1.01, 1.23)**	**1.11 (1.01, 1.23)**	**1.11 (1.01, 1.22)**	**1.11 (1.01, 1.22)**	**1.11 (1.01, 1.22)**
HOMA-β	1.08 (0.96, 1.21)	1.07 (0.95, 1.22)	1.07 (0.939, 1.23)	1.07 (0.943, 1.22)	1.08 (0.947, 1.23)

For FPG, the joint association represents the adjusted average change (in mg/dL) per quartile increase in metals; for insulin, HOMA-IR, and HOMA-β, the joint association represents the adjusted GMR per quartile increase in metals.

Model 1: adjusted for sex, maternal education, maternal socioeconomic status, exposure to environmental tobacco smoke defined by any smoker(s) in home at 4-year visit, age and season at outcome, and all other metals (n=422 participants for all outcomes; 1504, 706, 704, and 698 observations for FPG, insulin, HOMA-IR, and HOMA-β, respectively).
Model 2: adjusted for covariates in Model 1 plus zBMI at outcome (n=422 participants for all outcomes; 1502, 705, 703, and 697 observations for FPG, insulin, HOMA-IR, and HOMA-β, respectively).
Model 3: adjusted for covariates in Model 1 plus puberty status at outcome (n=420 participants for all outcomes; 779, 689, 687, and 681 observations for FPG, insulin, HOMA-IR, and HOMA-β, respectively).
Model 4: adjusted for covariates in Model 1 plus for diet (seafood, fast food, fruit and vegetable, and total energy intake) at 4-year visit (n=418 participants for all outcomes; 1490, 699, 697, and 691 observations for FPG, insulin, HOMA-IR, and HOMA-β, respectively).
Model 5: adjusted for covariates in Model 1 plus maternal characteristics (age and parity) (n=422 participants for all outcomes; 1504, 706, 704, and 698 observations for FPG, insulin, HOMA-IR, and HOMA-β, respectively).
